# TLR7 Protein Expression in Mild and Severe Lupus-Prone Models Is Regulated in a Leukocyte, Genetic, and IRAK4 Dependent Manner

**DOI:** 10.3389/fimmu.2019.01546

**Published:** 2019-07-10

**Authors:** Teja Celhar, Hao Kim Lu, Lia Benso, Larissa Rakhilina, Hui Yin Lee, Shubhita Tripathi, Olga Zharkova, Wei Yee Ong, Hiroko Yasuga, Bijin Au, Damien Marlier, Lina Hsiu Kim Lim, Thomas Paulraj Thamboo, John S. Mudgett, Matthew F. Mackey, Dennis M. Zaller, John E. Connolly, Anna-Marie Fairhurst

**Affiliations:** ^1^Singapore Immunology Network, A^*^STAR, Singapore, Singapore; ^2^Merck & Co., Inc., Boston, MA, United States; ^3^Institute of Molecular and Cell Biology, A^*^STAR, Singapore, Singapore; ^4^Department of Microbiology and Immunology, Yong Loo Lin School of Medicine, National University of Singapore, Singapore, Singapore; ^5^Department of Physiology, Yong Loo Lin School of Medicine, National University of Singapore, Singapore, Singapore; ^6^School of Biological Sciences, Nanyang Technological University, Singapore, Singapore; ^7^Tessa Therapeutics, Singapore, Singapore; ^8^Department of Pathology, National University Hospital, Singapore, Singapore; ^9^Institute of Biomedical Studies, Baylor University, Waco, TX, United States

**Keywords:** TLR7, IRAK4, anti-nuclear antibody, dendritic cells, SLE, lupus

## Abstract

The global increase in autoimmunity, together with the emerging autoimmune-related side effects of cancer immunotherapy, have furthered a need for understanding of immune tolerance and activation. Systemic lupus erythematosus (SLE) is the archetypical autoimmune disease, affecting multiple organs, and tissues. Studying SLE creates knowledge relevant not just for autoimmunity, but the immune system in general. Murine models and patient studies have provided increasing evidence for the innate immune toll like receptor-7 (TLR7) in disease initiation and progression. Here, we demonstrated that the kinase activity of the TLR7-downstream signaling molecule, interleukin-1 receptor associated kinase 4 (IRAK4), is essential for mild and severe autoimmune traits of the *Sle1* and *Sle1-TLR7* transgenic (*Sle1*Tg7) murine models, respectively. Elimination of IRAK4 signaling prevented all pathological traits associated with murine lupus, including splenomegaly with leukocyte expansion, detectable circulating antinuclear antibodies and glomerulonephritis, in both *Sle1* and *Sle1*Tg7 mice. The expansion of germinal center B cells and increased effector memory T cell phenotypes that are typical of lupus-prone strains, were also prevented with IRAK4 kinase elimination. Analysis of renal leukocyte infiltrates confirmed our earlier findings of an expanded conventional dendritic cell (cDC) within the kidneys of nephritic mice, and this was prevented with IRAK4 kinase elimination. Analysis of TLR7 at the protein level revealed that the expression in immune cells is dependent on the TLR7-transgene itself and/or autoimmune disease factors in a cell-specific manner. Increased TLR7 protein expression in renal macrophages and cDCs correlated with disease parameters such as blood urea nitrogen (BUN) levels and the frequency of leukocytes infiltrating the kidney. These findings suggest that controlling the level of TLR7 or downstream signaling within myeloid populations may prevent chronic inflammation and severe nephritis.

## Introduction

The immune system provides a crucial barrier against invasive pathogenic challenges. Defects can lead to opportunistic infections, cancer or the development of autoimmunity. Systemic lupus erythematosus (SLE) is the archetypical autoimmune disorder, whereby the immune system attacks itself in a systemic manner. It is complex and is driven by both genetic and environmental factors with no effective prevention or cure (1, 2).

SLE is characterized by the development of anti-nuclear antibodies (ANAs), particularly dsDNA- and RNA-reactive autoantibodies. These bind to self-antigens, resulting in the formation of immune complexes (ICs) which deposit in tissues, initiating, and promoting inflammation, tissue destruction, and often culminating in organ failure (3). The ICs in SLE patients contain U1 and Y1 RNA, with levels correlating with disease activity and the characteristic type-I interferon (IFN) signature (4). The primary candidate for RNA-associated IC activation is toll-like receptor (TLR)-7. TLR7 recognizes single-stranded RNA (ssRNA) and is central to host defense against invading viruses (5, 6). However, it also plays a fundamental role in the development and progression of autoimmunity. Immunological studies show that SLE sera can induce TLR7 expression in neutrophils and that these are primed for NETosis, which is also increased in SLE (7, 8). Furthermore, *TLR7* mRNA expression is higher in PBMCs from SLE patients, with levels correlating with the expression of IFNα (9).

Genetic studies have demonstrated *TLR7/TLR8* associations with SLE across multiple ethnicities (10, 11). The rs3853839 SNP in the 3′ untranslated region (3′ UTR) of *TLR7* is associated with SLE in the Asian population (11–13). The risk allele of rs3853839 confers higher levels of the *TLR7* transcript, most likely through an epigenetic microRNA mechanism (14). In a Mexican pediatric population, increased *TLR7* gene copy number was associated with higher levels of *TLR7* and IFNα mRNA (10). Since *TLR7* is located on the X chromosome, the gene dosage can be affected by aneuploidies. Indeed, both males and females with increased numbers of the X chromosome have an increased incidence of SLE (15, 16). Additionally, it has been recently shown that *TLR7* escapes from X chromosome inactivation, which results in a higher responsiveness to TLR7 ligands in females (17). This enhanced TLR7 expression likely contributes to the female bias in SLE and other autoimmune diseases.

Multiple murine models support a requirement for TLR7 in the development of ANAs and subsequent autoimmune pathology (18–20). A moderate increase of TLR7 through the BXSB-derived Y-autoimmune accelerator (*Yaa*) locus is essential for the rapid-onset of severe disease in *Yaa*-associated strains (21–24). Furthermore, we have shown that a ~2-fold upregulation of TLR7 in the mild NZM2410-derived *Sle1* background (*Sle1Tg7*) is sufficient for the progression to severe disease (25). In other model systems, a higher copy of TLR7 permits the progression to severe autoimmunity in the absence of any additional susceptibility locus (26).

The endosomal location of TLR7 is believed to be critical for the anti-viral response, in addition to preventing activation by self-RNA, which can lead to inflammation (27, 28). Stimulation with ssRNA activates a MyD88-dependent signaling cascade. In the early stages of activation, MyD88 recruits IL1R associated kinases (IRAK) 1, 2, and 4, forming the Myddosome (29). Phosphorylation of IRAK1 and IRAK2 leads to their dissociation from the complex and association and activation of TRAF6 and TAK1 (29–31). TAK1 activates MAPKs and subsequently triggers the activation of various transcription factors including interferon regulatory factors (IRF)3, IRF5, and IRF7, that have been previously described as risk factors for SLE (32–36), and NF-κB (29–31). This ultimately leads to the transcription and production of inflammatory cytokines and chemokines (37–39). The TAK1-dependent activation of NF-κB and IRF5 activation are mediated through the IRAK4 kinase domain (40). The IRAK4 kinase domain is necessary for the activation of IRF5 and for the TAK1-dependent activation of NF-κB. Additionally, IRAK4 also plays a structural, kinase-independent role, which can mediate TAK1-independent NF-κB activation (37, 41). The relative contribution of these pathways to the development of autoimmunity and severe inflammation is unclear.

A recent study investigated the role of IRAK4 in A20-binding inhibitor of NF-kB1 (ABIN1) mutant mice (42). ABIN1 is an ubiquitin sensing protein that interacts with death induced signaling complex (DISC) proteins to prevent FADD/caspase8 binding (43). Elimination of ABIN1 results in a TNF-dependent induction of programmed cell death, fetal liver apoptosis, anemia, hypoplasia, and early mortality (43). However, polyubiquitin-binding defective mutants of ABIN1 (ABIN1[D485N]) live longer, developing severe fibrosis in spleens, hearts, lungs, and intestine (44). These mice also develop murine lupus-like traits at a very young age (4 mos), which include splenomegaly with B cell and myeloid expansion, hypergammaglobulinemia with increases not only in IgG, but also in IgM, IgA and IgE, and ANAs (44). The ABIN1 mutant phenotypes are MyD88-dependent, possibly due to the loss of the humoral response and autoantibodies, as indicated in other models (19, 44). In addition, a mutation in IRAK1 or IRAK4 prevents the splenomegaly, ANA production, hypergammaglobulinemia and GN in ABIN1[D485N] mice (42). In a similar manner to the ABIN1 knockout, the phenotype is associated with TLR hyperactivation and increased TNF. However, it not known whether TNF reaches high systemic levels or if these lupus-traits are TNF dependent. Moreover, given the promiscuous nature of the activation of leukocytes, it is unclear as to the specific receptor dependency of disease and the role of IRAK4/1 on any given receptor. Furthermore, an assessment of leukocyte infiltration in the context of the diseased kidney would be helpful in understanding the regulatory roles of IRAK4 in this system.

Additionally, the role of TLR signaling and regulatory molecules was assessed in the BXSB lupus-prone model (45). The aggressive lupus-like phenotypes in the male are driven by a 2-fold increase in TLR7 expression due to the presence of the *yaa* locus (21, 23, 24). The females have normal TLR7 expression levels due to x-inactivation. Interestingly, Murphy et al. showed that female mice have increased expression of the TLR inhibitors, Tollip, and IRAK-M compared to their male *yaa*-expressing counterparts. In addition, the male mice had comparatively more phosphorylation following TLR4 or TLR7 ligation, despite having similar expression levels of IRAK1/4. Elimination of IRAK4 kinase activity in BXSB. IRAK4^ki/ki^ F2 mice resulted in a reduction in splenomegaly and number of splenic F4/80+ macrophages, neutrophils, total B cells, plasmablasts, and total CD11c^+^ cells (45). Supporting these findings, *in vivo* administration of a selective inhibitor of IRAK4, BMS-986126, prevented the development of GN, plasma anti-dsDNA autoantibodies and reduced IFNa^+^pDCs in both NZB/W and MRL^lpr^ mice, and splenic IL-6^+^CD11b^+^ myeloid cells in latter (46).

Taken together, these interesting investigations justify a more extensive analysis on leukocyte activation and expansion within the spleen itself, and in particular, the immunological events within the affected diseased kidney. Moreover, it is important to know if these suppressive effects of IRAK4 elimination on TLR7-dependent lupus-traits are due to the loss of susceptibility loci resulting from the introduction of B6-nonautoimmune background into the F2 progeny.

In these studies, we set out to characterize the role of IRAK4 kinase activity in the development of mild and severe TLR7-dependent systemic autoimmunity. In addition, we analyzed the renal leukocyte infiltrate and changes in protein levels of TLR7 in the context of pathology. We generated IRAK4 kinase dead (IRAK4^KD/KD^) mice and backcrossed these with *Sle1* and *Sle1*Tg7 mice to generate *Sle1*IRAK4^KD/KD^ and *Sle1*Tg7IRAK4^KD/KD^ mice. The model in our studies uses a low copy TLR7-BAC transgenic system, therefore we were able to examine genomic vs. disease effects on TLR7 protein expression in individual leukocyte compartments. Our data shows that IRAK4 kinase activity is essential for the development of all known autoimmune traits in both models, including splenomegaly, leukocyte activation, antibody production, and renal leukocyte recruitment with accompanying kidney disease. TLR7 is known to play a critical role in the onset of lupus, yet studies reporting comparative expression at the protein level are limited (47, 48). We assessed TLR7 protein levels using high dimensional flow cytometry and determined that the expression was regulated by disease and / or the low copy TLR7-BAC transgene in a leukocyte and tissue specific manner. Importantly, blood urea nitrogen (BUN) levels and CD45^+^ leukocyte infiltration, which are indicators of kidney inflammation and damage, correlated with TLR7 protein expression in renal cDCs and macrophages. Taken together our data demonstrates a complex cross-regulation between TLR7 receptor expression across multiple leukocytes that needs further investigation. Moreover, this work shows that the TLR7 downstream signaling pathway may be a key target for therapeutic intervention in lupus nephritis.

## Methods

### Mice

Mice were bred in the Biological Resource Center, A^*^STAR in Singapore or at Taconic in New York, USA. B6.*Sle1 (Sle1)* mice bearing NZM2410-lupus susceptibility intervals and the generation of the conditional BAC-Tg7 for increased TLR7 expression have been previously described (25, 49). Breeding pairs for TLR7^−/−^ mice were kindly provided by Prof. Shizuo Akira, Osaka University, Japan (5). The care and use of laboratory animals conformed to the NIH guidelines and all experimental procedures conformed to an IACUC approved animal protocol (#161176). Female mice were aged to 6–7 months to detect autoimmune traits.

### Generation of the K213A Construct/Mouse

The IRAK4 K213A point mutant mice were generated by Taconic Biosciences Inc. on behalf of Merck & Co., Inc., Boston, MA, USA. In brief, a BAC clone containing murine C57BL/6J genomic sequences was used to generate the targeting vector, which contains the K213A point mutation in exon 5 and an FRT site flanked puromycin resistance gene cassette in intron 5 for positive selection. Correctly targeted ES cell clones were injected into blastocysts and the resulting chimeras crossed to FLP deleted mice to remove the puromycin resistance gene cassette. Heterozygous mice were further bred to generate homozygous and wild-type control mice in a 100% C57BL/6 NTac genetic background for experiments. The level of IRAK4^KD/KD^ mRNA and protein expression in splenocytes of wild type, heterozygous, and homozygous mice was quantified using qPCR and Western Blotting, respectively. To confirm for the lack of IRAK4 kinase activity, GST-IRAK4^KD/KD^ or GST/TEV-IRAK4^WT/WT^ fusion proteins were generated and tested *in vitro* using an ULight kinase assay.

### Assessment of Renal Disease

Albumin in urine was quantified by ELISA (Abcam, Cambridge, UK) and the concentrations were normalized to urine creatinine measured with a colorimetric assay as per manufacturer's instructions (Cayman Chemical, Ann Arbor, MI). Blood Urea Nitrogen (BUN) was assessed using the QuantiChrom Urea Assay Kit (BioAssay Systems). To detect GN, kidneys were fixed in formalin and embedded in paraffin. Three micrometer sections of formalin-fixed, paraffin-embedded kidney tissues were stained with hematoxylin and eosin and with periodic acid–Schiff (PAS). Microscopic morphologic analysis was performed by an independent pathologist according to the International Society of Nephrology / Renal Pathology Society (ISN/RPS) 2003 classification of lupus nephritis (50); Class I, normal by light microscopy; Class II, mesangial proliferation; Class III, focal endocapillary or extracapillary proliferation, and/or subendothelial deposits; Class IV, diffuse endocapillary or extracapillary proliferation and/or subendothelial deposits; Class V, membranous lupus nephritis; Class VI, advanced sclerosing lupus nephritis.

### Serology

Sera were measured for circulating ANAs, dsDNA, anti-snRNP (1:200 dilution) and anti-RNA (1:100 dilution) using established ELISAs (25, 47, 51). HEp-2 staining was performed at a 1:200 dilution as per manufacturer's instructions (INOVA Diagnostics), with a goat-anti-mouse IgG-DyLight^®^488 secondary antibody (Abcam). HEp-2 were imaged at 100X using the Zeiss LSM 800 upright confocal microscope and processed with Zeiss Zen (Blue edition) software. HEp-2 staining patterns were evaluated by two independent investigators according to the International Consensus on Antinuclear Antibody (ANA) Patterns (ICAP). Luminex was used to quantify antibody subsets and subtypes including: IgA, IgM, IgG_1_, IgG_2a/c_, IgG_2b_, and IgG_3_ (Mouse Immunoglobulin Isotyping Magnetic Bead Panel, Merck Millipore). This panel is designed to detect IgG_2a_ (from BALB/c mice), which cross-reacts with IgG_2c_ from mice on the B6 background, which we have designated as IgG2_a/c_ (52).

### Fluorescent Microscopy

Frozen OCT embedded spleens were sectioned, fixed with 4% paraformaldehyde and stained with anti-CD4 (Ebioscience), anti-GL7 (BD Horizon) and anti-IgD (BD Pharmingen) antibodies. Confocal images of splenic germinal centers were collected as described above.

### Cell Preparation and Flow Cytometry

Single cells suspension from both spleen and kidney were prepared as described previously (53). Splenocytes or kidney cells were resuspended in PBS with 1% (vol/vol) FBS (FACs wash), counted and labeled with the following antibody panels. Splenic B cells: CD45, CD19, B220, CD138, CD21, CD23, GL7, Fas, CD11b, and CD11c. In some cohorts a more comprehensive B cell panel was used, including CD93 and IgM to identify transitional T1 (CD93^+^sIgM^hi^CD23^−^), T2 (CD93^+^sIgM^hi^CD23^+^), and T3 (CD93^+^sIgM^lo^CD23^+^) cells. Splenic T cells: CD45, CD3, CD4, CD8, CD44, CD62L, CD69, CXCR5, ICOS, and PD-1; splenic pDCs: CD11b, CD11c, mPDCA1, SiglecH, MHCII, CD86, CD9, CCR9, Gr-1, CD3, and B220. Splenic and kidney DCs & macrophages were stained with CD45, CD3, B220, CD8, CD11b, CD11c, Gr-1, F4/80, CD64, CD86, and MHCII. For the analysis of kidney B cells, T cells and pDCs and TLR7 expression in splenic and kidney subsets, the cells were stained with CD45, EpCAM, B220, CD44, CD62L, ICOS, CD138, CD4, CD8, NK1.1, Gr-1, mPDCA1, SiglecH, CD11b, CD11c, CD115, F4/80, MHC-II, and TLR7. All samples were stained with a live/dead fixable dye (Thermo Fisher Scientific, Waltham, MA). For intracellular TLR7 staining, the BD Cytofix/Cytoperm kit was used as per manufacturer instructions. Samples for the majority of panels were analyzed using a BD LSR Fortessa, a BD LSR II or BD Symphony with Flowjo 7.6. Antibodies were purchased from BD Biosciences, eBioscience, Biolegend, or Life Technologies.

### Statistical Analyses

Statistical analysis was performed with Graph Pad Prism 7.0. Normal distribution of the data was assessed by the Kolmogorov-Smirnov test. Data comparing the 4 strains was analyzed using a 1-way ANOVA followed by the Bonferroni's multiple comparison tests for normally distributed data, while the Kruskal-Wallis test with post hoc Dunn's multiple comparisons test was used for non-parametric data. *P* values < 0.05 were considered statistically significant. ^*^P < *0.05*, ^**^P < *0.01*, ^***^P < *0.001* and ^****^P < *0.0001* for ANOVA. Additional 2 group comparisons were completed for single gene changes where indicated, using a two-tailed Student's *t-test* (normal distribution) or Mann-Whitney *u-test* (non-parametric); ^#^*P* < *0.05*, ^##^*P* < *0.01*, ^###^*P* < *0.001* and ^####^*P* < *0.0001*. Correlations were assessed by computing Pearson's coefficient *r* and a two-tailed *t* test was used to establish if the correlations were significant (*P* < 0.05). For the comparison of survival curves, the log-rank (Mantel-Cox) test was performed and differences were considered statistically significant at *P* < 0.05 (^*^).

## Results

### Elimination of IRAK4 Kinase Activity Prevents Autoimmune Traits in Aged *Sle1* and *Sle1*Tg7 Mice

**Figure 1:**
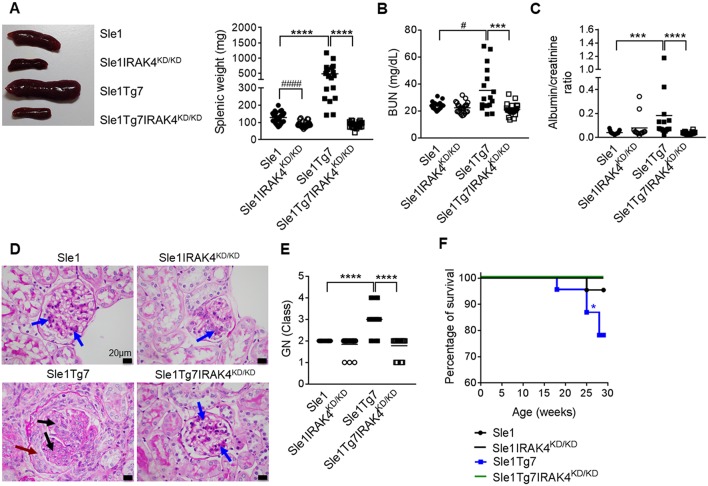
Prevention of spleen and kidney disease with the elimination of IRAK4 kinase activity. *Sle1, Sle1*IRAK4^KD/KD^, *Sle1*Tg7, and *Sle1*Tg7IRAK4^KD/KD^ female mice were aged to 6–7 months and analyzed for lupus disease parameters. **(A)** Photograph showing representative spleens from indicated strains and cumulative splenic weight (19–27 mice/group). Functional kidney assessment using **(B)** a blood urea nitrogen (BUN) assay in the same mice (17–23 mice/group) and **(C)** urine albumin levels normalized to creatinine (13–18 mice per group). Assessment of kidney disease class by PAS stain with **(D)** representative photomicrographs (original magnification x600, Black bars represent 20 μm), showing glomerular segments with mild mesangial proliferation (blue arrows), glomerular segments with endocapillary proliferation (black arrows) and cellular crescent (maroon arrow). **(E)** Cumulative data for kidney GN class for 17–19 mice/group (class I–IV represented as numbers 1–4). Parametric data were assessed by 1-way ANOVA (with Bonferroni's multiple comparisons test) and non-parametric data with Kruskal-Wallis (with Dunn's multiple comparisons test) ^***^P < 0.001, ^****^P < 0.0001. Additional comparisons between two groups were made with a Student's *t* test or Mann-Whitney test for parametric and non-parametric data, respectively, indicated by # (^#^*P* < 0.05, ^####^*P* < 0.0001). **(F)** Survival curves for each strain (22–23 mice per group); *Sle1*Tg7 mice survive significantly less than *Sle1*Tg7IRAK4^KD/KD^ (^*^P < 0.05, Mantel-Cox test).

We have previously shown that *Sle1*Tg7 mice develop severe autoimmune traits including splenomegaly and kidney disease at around 6 months of age (25, 51). In order to assess the role of IRAK4 kinase activity in the development of disease in this model, we generated IRAK4 kinase dead (IRAK4^KD/KD^) mice by engineering a mutation at position-213 from Lysine (K) to Alanine (A) using a plasmid with puromycin resistance as a selective marker (Figures S1A,B). This K213A IRAK4^KD/KD^ mutant was independently confirmed to lack kinase activity in an *in vitro* enzymatic assay (Figure S1C). We also confirmed comparable expression of IRAK4 mRNA and protein in IRAK4^KD/KD^ vs. wild-type splenocytes using qPCR and Western Blot, respectively (Figures S1D,E). We went on to breed these mice with *Sle1*Tg7 and aged 3 cohorts of female mice to ~6–7 months. Elimination of IRAK4 activity prevented both mild and severe splenomegaly exhibited by *Sle1* and *Sle1*Tg7 mice, respectively (Figure 1A). Analyses of kidney function showed expected increases in BUN levels and urine albumin concentration in *Sle1*Tg7 mice compared to *Sle1* controls, which were prevented in IRAK4 ^KD/KD^ mice (Figures 1B,C). Histopathology demonstrated that *Sle1*Tg7 kidneys had global endocapillary proliferation with cellular crescents in the glomeruli, while kidneys from *Sle1, Sle1*IRAK4^KD/KD^, and *Sle1*Tg7IRAK4^KD/KD^ mice showed only mild segmental mesangial proliferation (Figure 1D). Accordingly, the development of class 3–4 GN was completely prevented in *Sle1*Tg7IRAK4^KD/KD^ mice (Figure 1E). Consistent with these findings, the survival rate of *Sle1*Tg7 IRAK4^KD/KD^ mice was 100% at 6–7 months whilst only 78.3% *Sle1*Tg7 mice survived (Figure 1F).

### Circulating Autoantibodies and Immunoglobulin Titers Are Dependent on IRAK4 Kinase Activity

**Figure 2:**
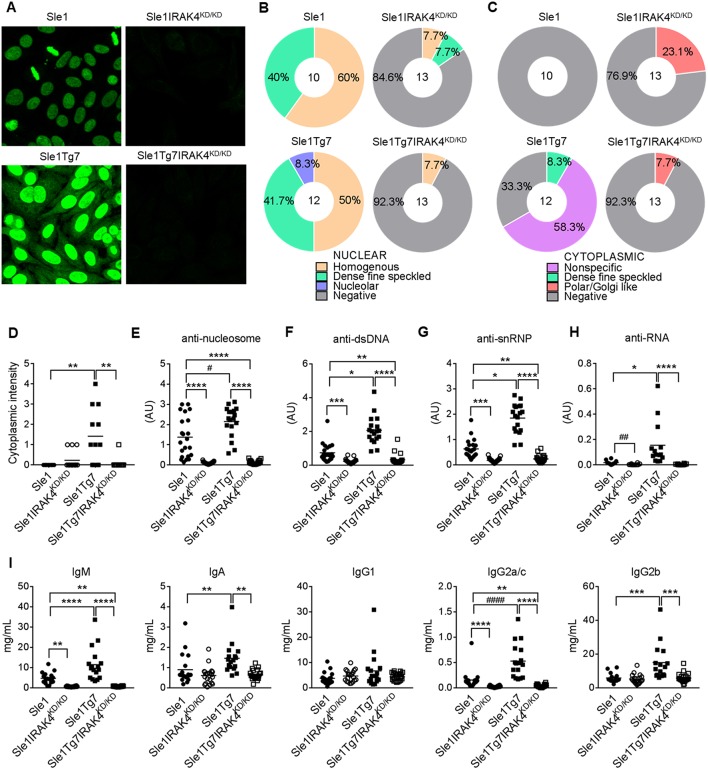
IRAK4 kinase activity is required for the production of autoantibodies and hypergammaglobulinemia observed in lupus-prone mice. **(A)** Representative microscopy images of HEp-2 staining patterns of serum autoantibodies from aged *Sle1, Sle1*IRAK4^KD/KD^, *Sle1*Tg7, and *Sle1*Tg7IRAK4^KD/KD^ mice. **(B,C)** Analysis of **(B)** nuclear and **(C)** cytoplasmic staining patterns determined by HEp-2 staining and microscopy. **(D)** Cytoplasmic intensity determined by HEp-2 microscopy. Serum for HEp-2 was from 3 independent cohorts with total 10–13 mice per group. **(E–H)** Analyses by ELISA of IgG autoantibodies with **(E)** nucleosome (dsDNA/histone), **(F)** dsDNA, or **(G)** snRNP or **(H)** RNA specificity in *Sle1, Sle1*IRAK4^KD/KD^, *Sle1*Tg7, and *Sle1*Tg7IRAK4^KD/KD^ mice (18–23 mice per group). **(I)** Luminex analysis of circulating antibody subclasses (IgM, IgA, IgG_1_, IgG_2a/c_, IgG_2b_, IgG_3_) across the 3 cohorts (data from 17–22 mice per group). Parametric data were assessed by 1-way ANOVA (with Bonferroni's multiple comparisons test) and non-parametric data with Kruskal-Wallis (with Dunn's multiple comparisons test) ^*^P < *0.05*, ^**^P < 0.01, ^***^P < 0.001, ^****^P < 0.0001. Additional comparisons between two groups were made with a Student's *t* test or Mann-Whitney test for parametric and non-parametric data respectively, indicated by # (^#^*P* < 0.05, ^##^*P* < 0.01, ^####^*P* < 0.0001).

Characterization of autoantibodies with HEp-2 staining and fluorescent microscopy showed the presence of anti-nuclear autoantibodies (ANAs) in *Sle1* mice, which were more pronounced in *Sle1*Tg7 mice, and prevented with IRAK4 kinase elimination (Figure 2A). Further analysis of HEp-2 staining patterns revealed an increase in the nucleolar and cytoplasmic reactive autoantibodies in *Sle1*Tg7 mice compared to controls (Figures 2B,D). The elimination of IRAK4 kinase activity resulted in Polar/Golgi-reactive antibodies in some *Sle1*IRAK4^KD/KD^ and *Sle1*Tg7IRAK4^KD/KD^ mice (Figure 2C), however their nature remains to be determined.

Our previously work has shown that *Sle1*Tg7 mice develop higher titers of anti-nucleosome, anti-dsDNA and anti-snRNP autoantibodies than their mild lupus-prone *Sle1* counterparts (25) (Figures 2E–G). In these studies we also found that anti-RNA autoantibodies were increased in *Sle1*Tg7 mice (Figure 2H). Elimination of IRAK4 kinase activity prevented the development of all autoantibodies examined in both *Sle1* and *Sle1*Tg7 mice (Figures 2E–H). Characterization of serum antibody isotypes showed increases in total IgM, IgA, IgG_2a/c_, and IgG_2b_ in *Sle1*Tg7 mice compared to *Sle1* controls and this was IRAK4 kinase dependent (Figure 2I). Interestingly IRAK4 was required for IgM and IgG2_a/c_ levels in *Sle1* mice, but not IgA or other IgG subtypes.

### IRAK4 Kinase Activity Is Essential for the Development of Splenic Autoimmune Traits Observed in Sle1 and *Sle1*Tg7 Mice

**Figure 3:**
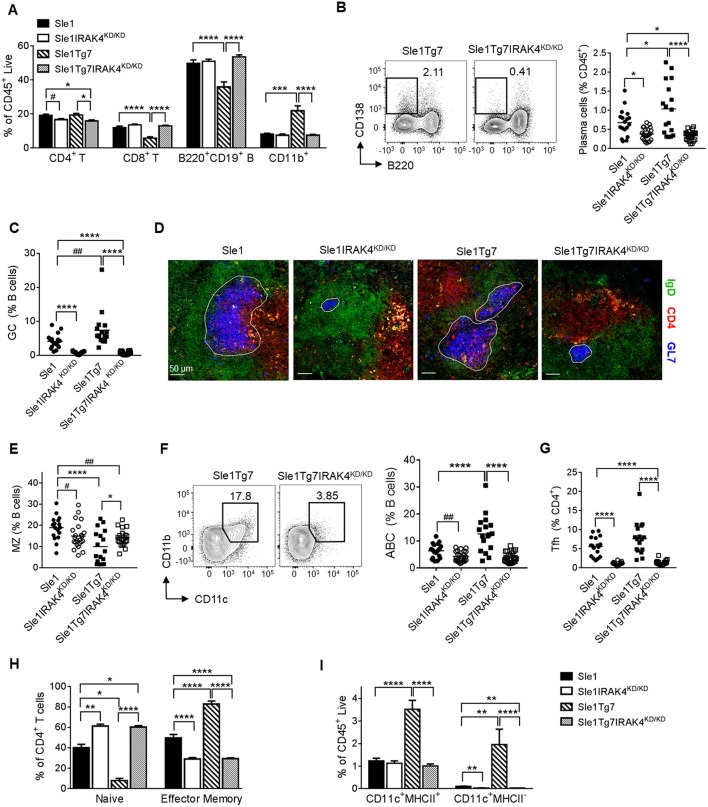
Functional elimination of IRAK4 prevents splenomegaly and associated leukocyte expansion observed in lupus-prone mice*. Sle1, Sle1*IRAK4^KD/KD^, *Sle1*Tg7, and *Sle1*Tg7IRAK4^KD/KD^ female mice were aged to 6–7 months and analyzed for characteristic autoimmune immunological traits. **(A)** Frequency of the indicated splenic leukocytes evaluated by flow cytometry. **(B)** Representative flow plots of plasma cells (CD138^+^B220^−^) from spleens of *Sle1*Tg7 and *Sle1*Tg7IRAK4^KD/KD^ mice with cumulative data from all strains. **(C)** GC B cell frequency measured by flow cytometry as GL7^+^Fas^+^ cells, represented as % B cells (CD19^+^B220^+^). **(D)** Representative immunofluorescent images of splenic GC staining, 300 x scale bar represents 50 μm, with anti-GL7 (blue), anti-CD4 (red) and anti-IgD antibodies (green). **(E)** Cumulative frequency of marginal zone (MZ) B cells (CD21^+^CD23^−^) across all strains (represented as % of CD19^+^B220^+^ B cells). **(F)** Representative flow plots of age-related B cells (CD11b^+^CD11c^+^) with cumulative data (represented as % of CD19^+^B220^+^ B cells). **(G)** Cumulative data of CD4^+^ CXCR5^+^PD1^+^ T follicular helper (T_FH_) cells, and **(H)** the frequencies of naïve (CD44^low^CD62L^high^) and effector memory (CD44^high^CD62L^low^) (represented as % of CD3^+^CD4^+^ T cells). **(I)** Frequencies of splenic CD11b^+^CD11c^+^MHC-II^+^ DCs and CD11b^+^CD11c^+^MHC-II^−^ cells. Flow cytometry data is from 17 to 23 mice per group. Parametric data were assessed by 1-way ANOVA (with Bonferroni's multiple comparisons test) and non-parametric data with Kruskal-Wallis (with Dunn's multiple comparisons test) ^*^P < *0.05*, ^**^P < 0.01, ^***^P < 0.001, ^****^P < 0.0001. Additional comparisons between two groups were made with a Student's *t* test or Mann-Whitney test for parametric and non-parametric data, respectively, indicated by # (^#^*P* < 0.05, ^##^*P* < 0.01).

Analyses of cell lineages using flow cytometry indicated that splenomegaly in *Sle1*Tg7 mice (Figure 1A) was characterized primarily by CD11b^+^ myeloid and CD4^+^ T cell expansion, as shown previously (Figure 3A and Table S1) (25, 51). This was prevented in IRAK4^KD/KD^ mice. Consistent with our antibody data (Figure 2), plasma cells and germinal center (GC) B cells were increased in *Sle1*Tg7 mice compared to *Sle1* controls and this was prevented with functional ablation of IRAK4 (Figures 3B,C). Moreover, the GC areas in *Sle1*IRAK4^KD/KD^ and *Sle1*Tg7IRAK4^KD/KD^ spleens were considerably smaller than in *Sle1* and *Sle1*Tg7 counterparts (Figure 3D). Therefore, we compared the GCs to age-matched non-autoimmune prone C57BL/6J (B6) mice and found them to be equivalent (Figures S2A,B). The decreased frequency of marginal zone (MZ) CD21^hi^CD23^−^ B cells identified previously in *Sle1*Tg7 and similar strains (25, 26, 51), was reduced but not completely restored with the elimination of IRAK4 kinase activity (Figure 3E). Additionally, we observed a higher percentage of age-related B cells (ABCs, B220^+^CD11c^+^CD11b^+^) in *Sle1*Tg7 mice compared to *Sle1* controls which was prevented in IRAK4^KD/KD^ mice (Figure 3F). Taken together, these finding suggest that the elimination of IRAK4 kinase activity prevents the progressive expansion, maturation and activation of B cells observed in the *Sle1*Tg7 strain.

Analyses of T cell subsets showed that CD4^+^ T follicular helper cells (T_FH_, CXCR5^+^ICOS^hi^) were fewer in IRAK4 ^KD/KD^ mice compared to either *Sle1* or *Sle1*Tg7 (Figure 3G). In a similar manner to the GCs, the T_FH_ frequencies in *Sle1*IRAK4^KD/KD^ spleens were comparable to non-autoimmune levels in B6 mice (Figure S2C). In addition, the increase in activation and expansion of effector memory CD4^+^ T cells characteristic of *Sle1*Tg7 was prevented in IRAK4^KD/KD^ mice (Figure 3H and Table S1) (25, 51). Analyses of CD8^+^ T cells revealed similar reductions in effector memory cells, with concomitant increases in the naïve population, in IRAK4^KD/KD^ mice (Table S1).

Analyses of myeloid subpopulations, cDCs, and CD8^+^ DCs were completed using the gating strategies in Figure S2D. The increase in the frequencies of eosinophils, PMNs, and both Gr1^+^ and Gr1^−^ CD11b^+^ cells in *Sle1*Tg7 mice were completely prevented by IRAK4 ^KD/KD^ (Figure S2E and Table S1). The increase observed in *Sle1*Tg7 mice in cDCs, their possible precursors (Gr1^−^CD11c^+^MHCII^−^) and the associated changes in MHCII and CD86 expression, were also prevented by IRAK4^KD/KD^ (Figure 3I, Figure S2F and Table S1. Analyses of CD8^+^ cDCs and plasmacytoid DCs (pDCs) frequencies showed a minor effect of disease or IRAK4^KD/KD^ (Figures S2G,H, including pDC gating strategy).

### Leukocyte Infiltration Associated With GN in *Sle1*Tg7 Mice Is Dependent on IRAK4 Kinase Activity

**Figure 4:**
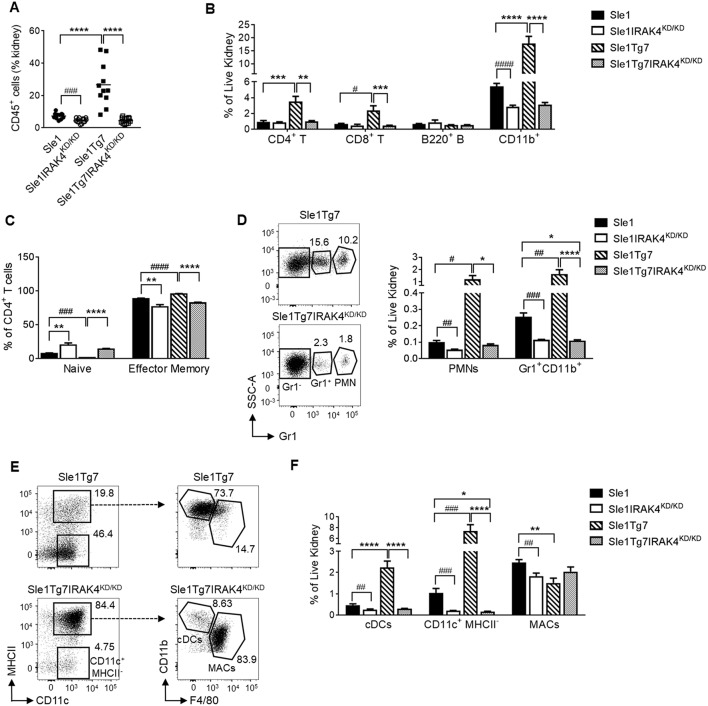
IRAK4 kinase activity is essential for renal leukocyte infiltration and activation of lupus prone mice. *Sle1, Sle1*IRAK4^KD/KD^, *Sle1*Tg7, and *Sle1*Tg7IRAK4^KD/KD^ female mice were aged to 6–7 months and analyzed for leukocyte infiltration and activation. Frequency of **(A)** total CD45^+^ leukocytes and **(B)** CD4^+^ T, CD8^+^ T, B220^+^ B and CD11b^+^ leukocyte subsets in the kidney by flow cytometry. **(C)** Cumulative data of the frequencies of naïve (CD44^low^CD62L^high^) and effector memory (CD44^high^CD62L^low^) CD4^+^ T cells. Representative flow plots and cumulative data of the frequencies of **(D)** PMNs and Gr-1^+^ CD11b^+^ myeloid cells and **(E)** CD11b^+^CD11c^+^MHC-II^+^ cDCs, CD11c^+^ MHCII^−^ cells and CD11b^+^CD11c^+^MHCII^+^F4/80^+^ macrophages (MACs) in the kidney. **(F)** Cumulative data of cells from **(E)** is shown. For **(A,C)** data from 11 to 14 mice/group is shown, while the rest is from 9 to 11 mice/group. Parametric data were assessed by 1-way ANOVA (with Bonferroni's multiple comparisons test) and non-parametric data with Kruskal-Wallis (with Dunn's multiple comparisons test) ^*^P < *0.05*, ^**^P < 0.01, ^***^P < 0.001, ^****^P < 0.0001. Additional comparisons between two groups were made with a Student's t test or Mann-Whitney test for parametric and nonparametric data respectively, indicated by # (^#^*P* < 0.05, ^##^*P* < 0.01, ^###^*P* < 0.001, ^####^*P* < 0.0001).

In a similar manner to our previous reports, we analyzed leukocyte infiltration into the kidney using flow cytometry (Figure S3A) (25, 51). We determined that the leukocyte expansion observed in *Sle1*Tg7 mice is dependent on IRAK4 kinase activity (Figures 4A,B). Moreover, CD4^+^ and CD8^+^ T cells from IRAK4^KD/KD^ mice consisted of fewer effector memory and more naïve populations (Figure 4C and Figure S3B. Further characterization of the myeloid population revealed that the increases observed in PMNs and Gr1^+^ CD11b^+^ cells in *Sle1*Tg7 kidneys were IRAK4 kinase dependent (Figure 4D). CD11b^+^Gr1^−^CD11c^+^ cells were gated for F4/80 and MHCII to characterize renal macrophages (F4/80^+^CD11c^+^MHCII^+^), CD11b^+^ cDCs (F4/80^−^CD11c^+^MHCII^+^) and their putative precursor (CD11c^+^MHCII^−^) (Figure 4E) (51). Consistent with our earlier investigations, both the CD11b^+^ cDC and cDC-precursor populations were expanded with severe disease in *Sle1*Tg7 mice (51), and this was prevented with IRAK4 ^KD/KD^ (Figures 4E,F). Furthermore, IRAK4 kinase elimination increased the expression of MHCII on DCs and macrophages (Figure S3C).

Additionally, we analyzed pDCs in the kidney using antibodies to B220, Siglec H and mPDCA1 (Figure S3A), which enabled a better identification than in our earlier study (51). We determined a significant increase of pDCs within the kidney, similar to previous observations (51) (Figure S3D).

### Splenic TLR7 Expression Is Regulated by Both the TLR7-BAC and Disease State in a Leukocyte-Specific Manner

We have previously shown effective detection of intracellular TLR7 protein using flow cytometry with clone A94B10 (47). We used a 17-color panel to investigate all major leukocyte subtypes in spleens and kidneys (gating strategy for splenocytes in Figure S4A). TLR7 expression was undetectable to low in CD4^+^ T cells, CD8^+^ T cells, NK1.1^+^ cells, PMNs, and CD8^+^ DCs as shown previously (Figures S4B–D and data not shown) (47).

**Figure 5:**
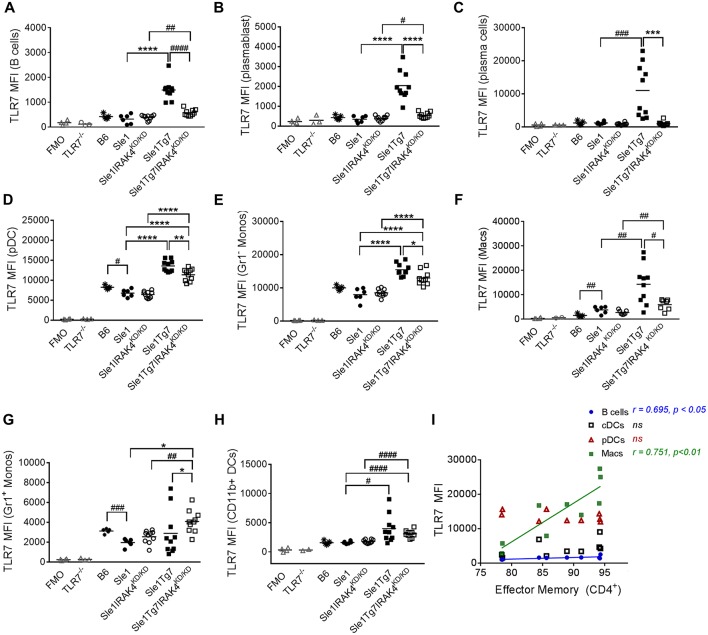
Expression of TLR7 in splenocytes is modulated by disease and Tg7 in a leukocyte specific manner. Splenocytes from 6 to 7months old B6, *Sle1, Sle1*Tg7, *Sle1*IRAK4^KD/KD^, and *Sle1*Tg7IRAK4^KD/KD^ female mice were analyzed for intracellular TLR7 expression using flow cytometry. TLR7^−/−^ mice and fluorescent minus one (FMO) controls were used to determine specificity. **(A)** Cumulative data of TLR7 expression in B cells, **(B)** plasmablasts, **(C)** plasma cells, and **(D)** CD115^+^Gr1^−^ monocytes (Gr1^−^ Monos), **(E)** F4/80^+^CD11b^lo^ macrophages (Macs), **(F)** B220^+^SiglecH^+^mPDCA1+ pDCs, **(G)** CD115^+^Gr1^+^ monocytes (Gr1^+^ Monos), **(H)** CD11b^+^CD11c^+^MHCII^+^ DCs (CD11b^+^DCs), across all strains. **(I)** Correlation of TLR7 expression in B cells, CD11b^+^ cDCs, pDCs and macrophages from *Sle1*Tg7 mice with effector memory CD4^+^ T frequencies in the spleen. Data from 6 to 10 mice per group is shown. Parametric data were assessed by 1-way ANOVA (with Bonferroni's multiple comparisons test) and non-parametric data with Kruskal-Wallis (with Dunn's multiple comparisons test); ^*^P < 0.05, ^**^P < 0.01, ^***^P < 0.001, ^****^P < 0.0001. Additional comparisons between two groups were made with a Student's *t* test or Mann-Whitney test for parametric and non-parametric data, respectively, indicated by # (^#^*P* < 0.05, ^##^*P* < 0.01, ^###^*P* < 0.001, ^####^*P* < 0.0001). Correlations were performed by calculating Pearson's correlation coefficients (r) and their significance assessed by a two-tailed *t*-test.

The mild autoimmunity of the *Sle1* mice had no effect on TLR7 in B cell subsets, as indicated by a comparison with the B6 control (Figures 5A–C). Although the expression was low in both B cells and plasmablasts of *Sle1* mice, levels were much higher in *Sle1*Tg7 mice (Figures 5A,B). This increase was largely prevented with the elimination of IRAK4 signaling. The fact that the elimination of IRAK4 kinase activity did not affect TLR7 levels in *Sle1* mice reduces the likelihood of this being a factor when comparing the *Sle1*Tg7 and Sle1Tg7IRAK^KD/^ ^KD^ mice. A comparison of *Sle1*IRAK^KD/KD^ and *Sle1*Tg7IRAK^KD/KD^ mice, which have similar phenotypes and pathologies, shows that the TLR7-BAC transgene (Tg7) increases TLR7 expression in both B cells and plasmablasts. Since expression in *Sle1*Tg7 is higher than the respective *Sle1*Tg7IRAK^KD/KD^ mice, it confirms that the inflammatory environment of severe autoimmunity and signaling through IRAK4 contributes to the increase in TLR7 expression (Figures 5A,B). Interestingly, TLR7 protein levels in plasma cells were only increased as a result of advanced autoimmunity and not the Tg7 itself (Figure 5C).

pDCs have been implicated in the development of SLE. Surprisingly, TLR7 levels were lower in pDCs from *Sle1* mice compared to B6 controls (Figure 5D). Furthermore, disruption of IRAK4 signaling did not affect TLR7 expression in these mild autoimmune mice. The increased expression in both *Sle1*Tg7 and *Sle1*Tg7IRAK4^KD/KD^ mice also demonstrates regulation of expression by both the BAC-Tg7 and the inflammatory milieu of severe pathology, congruent to B cell findings.

TLR7 levels in Gr^−^ (CD115^+^) monocytes and macrophages were augmented by the Tg7 and severe disease (Figures 5E,F). However, Gr1^+^ “inflammatory” monocyte TLR7 expression was only increased with the Tg7 (Figure 5G), independently of disease/signaling through IRAK4, with similar findings in CD11b^+^cDCs (Figure 5H). Interestingly, only macrophage TLR7 expression clearly correlated with CD4^+^ T cell effector memory frequency (Figure 5I). A positive association was also detected in B cells, however the biological relevance remains unclear, given the comparatively low TLR7 expression in this population.

### Kidney Leukocyte TLR7 Expression Is Regulated by the BAC, IRAK4, and Disease

**Figure 6:**
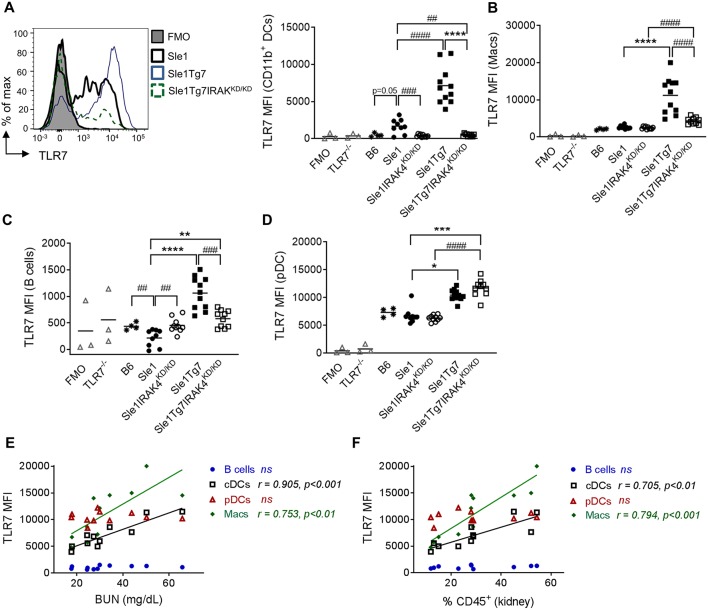
Expression of leukocytes in the kidney is modulated by disease and Tg7 transgene. Leukocytes in the kidney from 6 to 7 months old B6, *Sle1, Sle1*IRAK4^KD/KD^, *Sle1*Tg7, and *Sle1*Tg7IRAK4^KD/KD^ female mice were analyzed for intracellular TLR7 expression using flow cytometry. TLR7^−/−^ mice and fluorescent minus one (FMO) controls were used to determine specificity. **(A)** Representative overlay plots of intracellular TLR7 expression in CD11b^+^ DCs showing increases due to the transgenic and severe disease factors with cumulative data across all strains. Cumulative intracellular TLR7 protein expression in **(B)** CD11b^+^CD11c^+^F4/80^+^ macrophages (Macs), **(C)** B cells, **(D)** pDCs, **(E,F)** Correlation of TLR7 expression on B cells, CD11b^+^DCs (cDCs), pDCs, and macrophages (Macs) from *Sle1*Tg7 mice with **(H)** BUN and **(I)** percent of CD45 infiltration in kidney. Data from 9 to 11 mice per group is shown. Parametric data were assessed by 1-way ANOVA (with Bonferroni's multiple comparisons test) and non-parametric data with Kruskal-Wallis (with Dunn's multiple comparisons test) ^**^P < 0.01, ^***^P < 0.001, ^****^P < 0.0001. Additional comparisons between two groups were made with a Student's *t* test or Mann-Whitney test for parametric and non-parametric data, respectively, indicated by # (^##^*P* < 0.01, ^###^*P* < 0.001, ^####^*P* < 0.0001). Correlations were performed by calculating Pearson's correlation coefficients (r) and their significance assessed by a two-tailed *t*-test.

Our earlier studies have shown a critical role for renal CD11b^+^ cDCs in driving CD4^+^ T cell activation, inflammatory cytokine release and subsequent inflammation in *Sle1*Tg7 kidneys (51). In contrast to our results in the spleen, TLR7 levels in renal CD11b^+^ cDCs were observably higher in *Sle1* mice compared to B6 counterparts (*p* = 0.05; Figure 6A). In addition, the expression was further augmented with the increased disease severity of *Sle1*Tg7 mice (Figure 6A). Our data also showed that these disease associated increases in TLR7 were dependent on IRAK4 kinase activity. Given that our earlier findings showed that renal and splenic cDCs were of similar ontogeny (51), this suggests that the inflammatory environments play an enormous impact in TLR7 expression and there are significant differences between the largely immunological environment of the spleen and the mixed tissue and immune environment of the kidney.

Our analysis of renal macrophages and B cells showed increases in TLR7 as a result of severe disease (Figures 6B,C). Additionally, given the fact that IRAK4 kinase perturbation did not reduce TLR7 expression in renal *Sle1* macrophages, the increase in TLR7 in *Sle1*Tg7IRAK4^KD/KD^ compared to *Sle1*IRAK4^KD/KD^ may be attributed to the Tg7-BAC itself. However, since these mice have an increase in RNA-containing ICs, which provide a positive feedback loop of TLR7-Myddosome signaling, these assumptions may not be so simple.

In contrast to our findings in the spleen, TLR7 expression in kidney pDCs was dependent solely on the BAC-Tg7, since the elimination of IRAK kinase activity and disease had no effect (Figure 6D). The mixed cell populations, CD11b^+^Gr1^+^, CD11c^+^MHCII^−^ and CD11c^−^MHCII^−^ also showed similar gene and disease dependent changes (Figures S5A–C).

Additionally, we observed that TLR7 expression in renal CD11b^+^cDCs and macrophages, but not in pDCs and B cells, correlated with serum BUN and the total leukocyte infiltration (Figures 6E,F).

## Discussion

This report provides several advancements in our understanding of the immunological events occurring in autoimmunity using murine models of SLE. We conclusively demonstrated that the kinase domain of IRAK4 is necessary for all autoimmune traits in mild *Sle1* prone mice and in the TLR7-directed severe disease exhibited by the *Sle1*Tg7 model. Aside from Myddosome signaling, IRAK4 also plays a structural, kinase-independent role, which can mediate TAK1-independent NF-κB activation (37, 41). Our data and the work of others has effectively eliminated this as a major factor in the development of autoimmune traits (42, 45). Additionally, in this study, we comprehensively analyzed TLR7 protein expression across leukocyte subsets infiltrating the kidney, and in the spleen. We determined that TLR7 is regulated by the disease severity and IRAK4 kinase activity in a leukocyte and tissue specific manner. Importantly, we show that increased TLR7 protein in renal cDCs and macrophages correlates with measures of inflammation and kidney pathogenesis, pointing to their crucial role in the disease process.

Previous work has shown that IRAK4 phosphorylation is essential for the aggressive systemic autoimmunity and autoinflammation exhibited by the ABIN1 model (42). These mice develop key lupus phenotypes including ANA production and GN, however they also develop inflammation in tissues not commonly associated with clinical disease or exhibited by other lupus-prone murine models, such as liver and gut pathologies. Although many phenotypes can be attributed to increased TNFα signaling in the ABIN1 model, it seems unlikely that this is the mechanism of the lupus-like phenotypes (44). Congruent to this, we have shown that TNFα levels, even at the site of the diseased kidney, are not affected by systemic increases in TLR7 or the severe autoimmune environment (25). The ABIN1 studies also show that, despite the upregulation of type I IFNs, elimination of IFNAR1 only partially reduced the development of GN, and did not affect other phenotypes (42). Therefore, the cytokines and critical cells mediating the pathologies remain elusive. The widespread hyperactivation of the TLR family, together with BCR signaling suggests a more complicated mechanism of disease in this model. We and others have suggested a stepwise progression of the development of polygenic associated lupus (54, 55). The initial steps in disease development confer a loss of tolerance, the generation of autoreactive T and B cells and autoantibody development. The progression to severe disease requires an additional immune alteration, which in many cases has been attributed to innate dysregulation. Given the retention of autoimmune phenotypes following backcrossing to the B6 background by Cohen and colleagues, it is clear that this single mutation of ABIN1 mediates dysregulation of both immune tolerance and innate activation (42, 44). Certainly, the loss of MyD88, and disrupted IRAK1/4 signaling points to the essential nature of the TLR and IL1R families for the mechanism of aggressive disease.

In our studies we were able to analyse the contribution of IRAK4-mediated signaling at different stages of disease. We and others have used B6.*Sle1* mice as an example of step 1 in the pathway to severe disease, given their mild phenotypes and that they rarely develop GN in the absence of an additional susceptibility locus (24, 47, 51, 56). The *sle1* susceptibility locus on chromosome 1 is shared across most spontaneous murine model systems (54). Previous work by Wakeland et al. has shown that the NZM2410-derived *sle1* phenotypes are conferred by polymorphisms in the SLAMF members, Ly108, CD82, CD244, CD229, and CD319 (57, 58). Our data shows that IRAK4 phosphorylation is required for the break in tolerance to nuclear antigens and ANA production in *Sle1* mice. In addition, all mild autoimmune traits exhibited by these mice appear to be prevented. The reductions in splenic B and T cell activation and expansion are akin to the B6 phenotypes, suggesting that MyD88/IRAK pathway disruption is targeting spontaneous autoimmune mechanisms, rather that core developmental processes. Our data also suggests that while the MyD88/IRAK pathway is critical for these *Sle1*-conferred phenotypes, changes in TLR7 at the protein level do not play a significant role. In addition, it is likely that the loss in tolerance mediated by the SLAMF polymorphisms in *Sle1* mice develops with the assistance of other TLR/IL1R family members, given the promiscuous nature of the Myddosome and IRAK4 kinase activity.

To examine severe disease, we used our low copy TLR7 BAC transgenic on the *Sle1* background, *Sle1*Tg7. These mice develop similar phenotypes to the BXSB strain without the male dependency for GN. Therefore, cohorts of the same sex or comparisons between sexes can be analyzed, allowing for sex-independent or sex-dependent immunological pathway analysis. Furthermore, *Sle1* mice have a female sex bias similar to clinical disease, most likely attributed to the incomplete X inactivation, or the XX complex itself (59, 60).

An additional advantage of using B6.*Sle1*/*Sle1*Tg7 mice was the retention of the same non-autoimmune background (B6), eliminating influences of other loci. In these studies, we were able to confirm that the elimination of proteinuria and splenomegaly seen in BXSB*Yaa*.IRAK4^KD/KD^ mice was not due to the dilution of susceptibility loci present in the BXSB background by the introduction of the B6 non-autoimmune genome (45). However, in contrast to the data from Murphy et al. our heterozygous *Sle1*Tg7IRAK4^WT/KD^ mice developed severe disease in a similar manner to *Sle1*Tg7IRAK4^KD^/IRAK4^KD^ homozygotes (data not shown). The increased disease in the BXSB F2 heterozygous highlights additional loci which contribute to disease and are not present in the B6 background of *Sle1*Tg7 mice. Similar to the findings of the homozygous BXSB*Yaa*.IRAK4^KD/KD^ male mice shown in the above study, we saw a prevention in the expansion of B cells and plasma cells, with IRAK4 kinase elimination. The splenomegaly by most spontaneous severe lupus models is also characterized by increases in germinal center B cells and T follicular helper cells, together with T cell activation (25, 26, 51). We were also able to show that these changes were prevented with IRAK4 kinase perturbation. A more in-depth analysis of the CD11c^+^ lineage excluded neutrophils and revealed that CD11b^+^ cDCs and their putative precursors, CD11c^+^MHC^−^ cells, comprised the majority of this expansion. Our splenic analysis also showed that in the majority of leukocytes, TLR7 was upregulated not only through the genomic contribution of the Tg7-BAC, but also by the intense autoimmune environment observed in the *Sle1*Tg7 mice.

Our work also provides a detailed analysis of the immunological events occurring not only in the spleen, but also in the diseased kidney. Approximately 50% of SLE patients develop GN and obvious challenges restrict comprehensive understanding of the inflammatory events occurring. Given the burgeoning data on the role of TLR7 in SLE pathogenesis, analysis at the protein level within the tissue may prove to be invaluable. Overall IRAK4 kinase ablation prevented both mild and severe kidney disease in the *Sle1* and *Sle1*Tg7 models, respectively, together with the associated T cell and DC infiltrate. Our examination of TLR7 protein levels using flow cytometry showed that the autoimmune inflammatory environment was able to augment TLR7 expression, with the exception of renal infiltrating pDCs. Moreover, renal CD11b^+^cDC TLR7 expression was associated with key disease parameters, including BUN levels and the renal leukocyte infiltrate. This may suggest that the development of a soluble TLR7 assay as a urine biomarker could prove to be clinically relevant. However, given the endosomal location of TLR7, it is unclear as to whether this is possible and therefore warrants further investigation.

Previous data from a number of groups has shown that TLR7 plays a critical role in the initial stages of autoimmunity and development of ANAs (18–20). Interestingly, elimination of TLR7 in murine SLE results in similar phenotypes to our IRAK4 kinase deficient *Sle1* mice, including a decreased GC formation, splenic T_FH_ frequencies and serum autoantibody titers (18, 19, 61, 62). In addition, elimination of the downstream transcription factor, IRF5 in MRL^lpr^ lupus prone mice results in a similar ablation of all autoimmune phenotypes (63). In patient genome wide association studies (GWAS), IRF5 polymorphisms have also associated with disease development (33–36). Congruent to these findings, the elimination of IRAK4 signaling prevents TLR-induced IKKβ phosphorylation and the subsequent translocation of the transcription factor IRF5 to the nucleus (40). This prevents the TLR induced (and Myd88 dependent) cytokine production. At the same time, the Myddosome complex becomes more stable and NFβB nuclear translocation remains intact (40, 64). Taken together these findings indicate that the autoimmunity observed across multiple model systems is dependent on a TLR7, MyD88-IRAK4 kinase, and IRF5 dependent signaling cascade, which is independent of NFkB nuclear translocation and cytokine production. Given that the region encompassing *Sle1* on chromosome is a common susceptibility locus, additionally identified in NZB/W and in MRL^lpr^ mice, and previous work showing TLR7 and MyD88 elimination preventing autoimmune phenotypes, we propose that IRAK kinase perturbations in these polygenic murine models would result in eliminating autoimmunity. Further studies into the signaling, nuclear translocation and cytokine production would shed further light on these mechanisms, particularly given the bias toward TLR7 of our model system.

We and others have previously shown that IFNα and TLR7 ligands, such as RNA-containing viruses and TLR7 agonist R848 can increase TLR7 mRNA expression (51, 65). Since RNA-containing immune complexes also act as TLR7 ligands (66), they may increase TLR7 expression in lupus models and clinical disease through a positive feedback loop. Several studies support this hypothesis: SLE serum increases TLR7 expression (7) and SLE patients with an anti-RNA-associated antibody profile have preferentially higher TLR7 expression (67). TLR7 ligand hyper-responsiveness has been identified as the main driver of lupus-like pathology in pristane-induced mouse models of SLE (48, 68). Our studies in TLR9-deficient mice have also attributed the increase in TLR7 protein expression to an anti-RNA antibody profile and severe disease (47). In the present study we have shown that disease can directly contribute to the increase of TLR7 expression in various cell types. Eliminating downstream TLR7 (and other TLR) signaling through IRAK4 by using IRAK4 kinase deficient mice prevents the development of disease and TLR7 upregulation beyond the effects of the BAC. Blocking signal transduction and preventing TLR7 upregulation by inhibiting IRAK4 thus represents a possible therapeutic target for SLE treatment.

Over 90 patents for small molecule IRAK4 inhibitors have been filed from 2016 to 2018, primarily for autoimmune diseases and cancer (46, 69). In keeping with the data from lupus mice shown here and by others (42, 45), the IRAK4 inhibitor BMS-986126 suppressed the development of lupus in the NZB/NZW and MRL^*lpr*^ models (46). Similarly, IRAK4 inhibitors, ND-2158 and ND-2110 attenuated TNFα production from TLR4/9-stimulated human PBMCs and autoimmune disease in collagen-induced arthritis and gout murine models (70). In separate studies relating to SLE pathogenesis, IRAK4 inhibitor, ND-2158 reduced cytokine production by human plasmacytoid dendritic cells and natural killer cells stimulated by RNA- ICs (71). However, in these studies did not find an increase in TNF or IFNα production in SLE PBMCs compared to controls, perhaps due to medication or leukocyte exhaustion. Given these findings and the broad role of IRAK4, it is unclear as to whether the toxicity of the side effects will prove too limiting for clinical use (72).

In summary, the findings in our study show a dependency on IRAK4 kinase activity for increased TLR7 protein levels, together with mild and severe autoimmune phenotypes of the *Sle1* and *Sle1*Tg7 models, respectively. The regulation of TLR7 expression is not as simple as one expects, and depends on the pathogenic environment and the individual leukocyte involved. Further studies are required to further elucidate these factors, including cytokine induced nascent expression of TLR7 which may exacerbate BAC-induced TLR7 expression.

Taken together, our findings show the importance of increased signaling through TLR7 in SLE and targeting this pathway, may lead to a suppression of severe disease development.

## Ethics Statement

This study was carried out in accordance with the recommendations of the Biological Resource Center of A^*^STAR. The protocol was approved by the IACUC committee, protocol ID 161176.

## Author Contributions

TC, HKL, HYL, ST, OZ, WYO, HY, LB, LR, BA, TPT, and A-MF performed the experiments. TC, HKL, DM, LB, LR, TBT, and A-MF analysed the data. A-MF, TC, and HKL wrote the manuscript. A-MF, DMZ, JSM, and JEC designed the research studies. A-MF, DMZ, MFM, JSM, JEC, and LHKL provided reagents, mice and/or statistical support.

### Conflict of Interest Statement

The authors declare that this study received funding through a joint innovation grant between Merck & Co., Inc. and A*STAR, Singapore. LB and LR are employees of Merck. MFM, JSM, and DMZ were employees of Merck at the time of the study. DM was an employee of Tessa at the time of the study. The remaining authors declare that the research was conducted in the absence of any commercial or financial relationships that could be construed as a potential conflict of interest.

## References

[1] Kaul A Gordon C Crow MK Touma Z Urowitz MB van Vollenhoven R. Systemic lupus erythematosus. Nat Rev Dis Primers. (2016) 2:16039. 10.1038/nrdp.2016.40 27306639

[2] Moser KL Kelly JA Lessard CJ Harley JB. Recent insights into the genetic basis of systemic lupus erythematosus. Genes Immun. (2009) 10:373–9. 10.1038/gene.2009.39 19440199 PMC3144759

[3] Mayadas TN Tsokos GC Tsuboi N. Mechanisms of immune complex-mediated neutrophil recruitment and tissue injury. Circulation. (2009) 120:2012–24. 10.1161/CIRCULATIONAHA.108.771170 19917895 PMC2782878

[4] Doedens JR Jones WD Hill K Mason MJ Gersuk VH Mease PJ l. Blood-Borne RNA correlates with disease activity and IFN-stimulated gene expression in systemic lupus erythematosus. J Immunol. (2016) 197:2854–63. 10.4049/jimmunol.1601142 27534558

[5] Hemmi H Kaisho T Takeuchi O Sato S Sanjo H Hoshino K. Small anti-viral compounds activate immune cells via the TLR7 MyD88-dependent signaling pathway. Nat Immunol. (2002) 3:196–200. 10.1038/ni758 11812998

[6] Lund JM Alexopoulou L Sato A Karow M Adams NC Gale NW. Recognition of single-stranded RNA viruses by Toll-like receptor 7. Proc Natl Acad Sci USA. (2004) 101:5598–603. 10.1073/pnas.0400937101 15034168 PMC397437

[7] Garcia-Romo GS Caielli S Vega B Connolly J Allantaz F Xu Z. Netting neutrophils are major inducers of type I IFN production in pediatric systemic lupus erythematosus. Sci Transl Med. (2011) 3:73ra20. 10.1126/scitranslmed.3001201 21389264 PMC3143837

[8] Lood C Arve S Ledbetter J Elkon KB. TLR7/8 activation in neutrophils impairs immune complex phagocytosis through shedding of FcgRIIA. J Exp Med. (2017) 214:2103–19. 10.1084/jem.20161512 28606989 PMC5502427

[9] Komatsuda A Wakui H Iwamoto K Ozawa M Togashi M Masai R. Up-regulated expression of Toll-like receptors mRNAs in peripheral blood mononuclear cells from patients with systemic lupus erythematosus. Clin Exp Immunol. (2008) 152:482–7. 10.1111/j.1365-2249.2008.03646.x 18373699 PMC2453201

[10] García-Ortiz H Velázquez-Cruz R Espinosa-Rosales F Jiménez-Morales S Baca V Orozco L. Association of TLR7 copy number variation with susceptibility to childhood-onset systemic lupus erythematosus in Mexican population. Ann Rheum Dis. (2010) 69:1861–5. 10.1136/ard.2009.124313 20525845

[11] Lee YH Choi SJ Ji JD Song GG. Association between toll-like receptor polymorphisms and systemic lupus erythematosus: a meta-analysis update. Lupus. (2016) 25:593–601. 10.1177/0961203315622823 26762473

[12] Kawasaki A Furukawa H Kondo Y Ito S Hayashi T Kusaoi M. TLR7 single-nucleotide polymorphisms in the 3' untranslated region and intron 2 independently contribute to systemic lupus erythematosus in Japanese women: a case-control association study. Arthritis Res Ther. (2011) 13:R41. 10.1186/ar3277 21396113 PMC3132023

[13] Shen N Fu Q Deng Y Qian X Zhao J Kaufman KM. Sex-specific association of X-linked Toll-like receptor 7 (TLR7) with male systemic lupus erythematosus. Proc Natl Acad Sci USA. (2010) 107:15838–43. 10.1073/pnas.1001337107 20733074 PMC2936646

[14] Deng Y Zhao J Sakurai D Kaufman KM Edberg JC Kimberly RP. MicroRNA-3148 modulates allelic expression of toll-like receptor 7 variant associated with systemic lupus erythematosus. PLoS Genet. (2013) 9:e1003336. 10.1371/journal.pgen.1003336 23468661 PMC3585142

[15] Dillon SP Kurien BT Li S Bruner GR Kaufman KM Harley JB Sex chromosome aneuploidies among men with systemic lupus erythematosus. J Autoimmun. (2012) 38(2–3):J129–34. 10.1016/j.jaut.2011.10.004 22154021 PMC3309073

[16] Liu K Kurien BT Zimmerman SL Kaufman KM Taft DH Kottyan LC. X chromosome dose and sex bias in autoimmune diseases: increased prevalence of 47,XXX in systemic lupus erythematosus and sjogren's syndrome. Arthritis Rheumatol. (2016) 68:1290–1300. 10.1002/art.39560 26713507 PMC5019501

[17] Souyris M Cenac C Azar P Daviaud D Canivet A Grunenwald S. TLR7 escapes X chromosome inactivation in immune cells. Sci Immunol. (2018) 3:eaap8855. 10.1126/sciimmunol.aap8855 29374079

[18] Berland R Fernandez L Kari E Han JH Lomakin I Akira S. Toll-like receptor 7-dependent loss of B cell tolerance in pathogenic autoantibody knockin mice. Immunity. (2006) 25:429–40. 10.1016/j.immuni.2006.07.014 16973388

[19] Nickerson KM Christensen SR Shupe J Kashgarian M Kim D Elkon K. TLR9 regulates TLR7- and MyD88-dependent autoantibody production and disease in a murine model of lupus. J Immunol. (2010) 184:1840–8. 10.4049/jimmunol.0902592 20089701 PMC4098568

[20] Santiago-Raber ML Dunand-Sauthier I Wu T Li QZ Uematsu S Akira S. Critical role of TLR7 in the acceleration of systemic lupus erythematosus in TLR9-deficient mice. J Autoimmun. (2010) 34:339–48. 10.1016/j.jaut.2009.11.001 19944565

[21] Pisitkun P Deane JA Difilippantonio MJ Tarasenko T Satterthwaite AB Bolland S. Autoreactive B cell responses to RNA-related antigens due to TLR7 gene duplication. Science. (2006) 312:1669–72. 10.1126/science.1124978 16709748

[22] Fairhurst AM Hwang SH Wang A Tian XH Boudreaux C Zhou XJ. Yaa autoimmune phenotypes are conferred by overexpression of TLR7. Eur J Immunol. (2008) 38:1971–8. 10.1002/eji.200838138 18521959 PMC2993003

[23] Santiago-Raber ML Kikuchi S Borel P Uematsu S Akira S Kotzin BL. Evidence for genes in addition to Tlr7 in the Yaa translocation linked with acceleration of systemic lupus erythematosus. J Immunol. (2008) 181:1556–62. 10.4049/jimmunol.181.2.1556 18606711

[24] Subramanian S Tus K Li QZ Wang A Tian XH Zhou J. A Tlr7 translocation accelerates systemic autoimmunity in murine lupus. Proc Natl Acad Sci USA. (2006) 103:9970–5. 10.1073/pnas.0603912103 16777955 PMC1502563

[25] Hwang SH Lee H Yamamoto M Jones LA Dayalan J Hopkins R. B cell TLR7 expression drives anti-RNA autoantibody production and exacerbates disease in systemic lupus erythematosus-prone mice. J Immunol. (2012) 189:5786–96. 10.4049/jimmunol.1202195 23150717 PMC3544945

[26] Deane JA Pisitkun P Barrett RS Feigenbaum L Town T Ward JM. Control of toll-like receptor 7 expression is essential to restrict autoimmunity and dendritic cell proliferation. Immunity. (2007) 27:801–10. 10.1016/j.immuni.2007.09.009 17997333 PMC2706502

[27] Lee BL Barton GM. Trafficking of endosomal Toll-like receptors. Trends Cell Biol. (2014) 24:360–9. 10.1016/j.tcb.2013.12.002 24439965 PMC4037363

[28] Mouchess ML Arpaia N Souza G Barbalat R Ewald SE Lau L. Transmembrane mutations in Toll-like receptor 9 bypass the requirement for ectodomain proteolysis and induce fatal inflammation. Immunity. (2011) 35:721–32. 10.1016/j.immuni.2011.10.009 22078797 PMC3230302

[29] Li X. IRAK4 in TLR/IL-1R signaling: possible clinical applications. Eur J Immunol. (2008) 38:614–8. 10.1002/eji.200838161 18286571

[30] Beutler B. Microbe sensing, positive feedback loops, and the pathogenesis of inflammatory diseases. Immunol Rev. (2009) 227:248–63. 10.1111/j.1600-065X.2008.00733.x 19120489 PMC2713013

[31] Kawai T Akira S. Toll-like receptors and their crosstalk with other innate receptors in infection and immunity. Immunity. (2011) 34:637–50. 10.1016/j.immuni.2011.05.006 21616434

[32] Fu Q Zhao J Qian X Wong JL Kaufman KM Yu CY. Association of a functional IRF7 variant with systemic lupus erythematosus. Arthritis Rheum. (2011) 63:749–54. 10.1002/art.30193 21360504 PMC3063317

[33] Harley JB Alarcón-Riquelme ME Criswell LA Jacob CO Kimberly RP. Genome-wide association scan in women with systemic lupus erythematosus identifies susceptibility variants in ITGAM, PXK, KIAA1542 and other loci. Nat Genet. (2008) 40:204–10. 10.1038/ng.81 18204446 PMC3712260

[34] Kelly JA Kelley JM Kaufman KM Kilpatrick J Bruner GR Merrill JT. Interferon regulatory factor-5 is genetically associated with systemic lupus erythematosus in African Americans. Genes Immun. (2008) 9:187–94. 10.1038/gene.2008.4 18288123

[35] Niewold TB Kelly JA Flesch MH Espinoza LR Harley JB Crow MK. Association of the IRF5 risk haplotype with high serum interferon-alpha activity in systemic lupus erythematosus patients. Arthrit Rheum. (2008) 58:2481–7. 10.1002/art.23613 18668568 PMC2621107

[36] Sigurdsson S Nordmark G Garnier S Grundberg E Kwan T Nilsson O. A risk haplotype of STAT4 for systemic lupus erythematosus is over-expressed, correlates with anti-dsDNA and shows additive effects with two risk alleles of IRF5. Hum Mol Genet. (2008) 17:2868–76. 10.1093/hmg/ddn184 18579578 PMC2525501

[37] Fraczek J Kim TW Xiao H Yao J Wen Q Li Y. The kinase activity of IL-1 receptor-associated kinase 4 is required for interleukin-1 receptor/toll-like receptor-induced TAK1-dependent NFkappaB activation. J Biol Chem. (2008) 283:31697–705. 10.1074/jbc.M804779200 18794297 PMC2581573

[38] Wang JQ Jeelall YS Ferguson LL Horikawa K. Toll-like receptors and cancer: MYD88 mutation and inflammation. Front Immunol. (2014) 5:367. 10.3389/fimmu.2014.00367 25132836 PMC4116802

[39] Zhu J Mohan C. Toll-like receptor signaling pathways–therapeutic opportunities. Mediators Inflamm. (2010) 2010:781235. 10.1155/2010/781235 20981241 PMC2963142

[40] Cushing L Winkler A Jelinsky SA Lee K Korver W Hawtin R. IRAK4 kinase activity controls Toll-like receptor induced inflammation through the transcription factor IRF5 in primary human monocytes. J Biol Chem. (2017) 292:18689–98. 10.1074/jbc.M117.796912 28924041 PMC5682975

[41] Lye E Mirtsos C Suzuki N Suzuki S Yeh WC. The role of interleukin 1 receptor-associated kinase-4 (IRAK-4) kinase activity in IRAK-4-mediated signaling. J Biol Chem. (2004) 279:40653–8. 10.1074/jbc.M402666200 15292196

[42] Nanda SK Lopez-Pelaez M Arthur JS Marchesi F Cohen P Suppression of IRAK1 or IRAK4 catalytic activity, but not Type 1 IFN signaling, prevents lupus nephritis in mice expressing a Ubiquitin binding-defective mutant of ABIN1. J Immunol. (2016) 197:4266–73. 10.4049/jimmunol.1600788 27807192 PMC5114882

[43] Oshima S Turer EE Callahan JA Chai S Advincula R Barrera JOshima S, et al. ABIN-1 is a ubiquitin sensor that restricts cell death and sustains embryonic development. Nature. (2009) 457:906–9. 10.1038/nature07575 19060883 PMC2642523

[44] Nanda SK Venigalla RK Ordureau A Patterson-Kane JC Powell DW Toth R. Polyubiquitin binding to ABIN1 is required to prevent autoimmunity. J Exp Med. (2011) 208:1215–28. 10.1084/jem.20102177 21606507 PMC3173241

[45] Murphy M Pattabiraman G Manavalan TT Medvedev AE. Deficiency in IRAK4 activity attenuates manifestations of murine Lupus. Eur J Immunol. (2017) 47:880–91. 10.1002/eji.201646641 28295231 PMC5575829

[46] Dudhgaonkar S Ranade S Nagar J Subramani S Prasad DS Karunanithi P. Selective IRAK4 inhibition attenuates disease in murine lupus models and demonstrates steroid sparing activity. J Immunol. (2017) 198:1308–19. 10.4049/jimmunol.1600583 28003376 PMC5253435

[47] Celhar T Yasuga H Lee HY Zharkova O Tripathi S Thornhill SI TLR9 deficiency breaks tolerance to RNA-associated antigens and upregulates TLR7 protein in Sle1 mice. Arthritis Rheumatol. (2018) 70:1597–609. 10.1002/art.40535 29687651 PMC6175219

[48] Bossaller L Christ A Pelka K Nündel K Chiang PI Pang C. TLR9 Deficiency leads to accelerated renal disease and myeloid lineage abnormalities in pristane-induced murine lupus. J Immunol. (2016) 197:1044–53. 10.4049/jimmunol.1501943 27354219 PMC5266544

[49] Morel L Mohan C Yu Y Croker BP Tian N Deng A. Functional dissection of systemic lupus erythematosus using congenic mouse strains. J Immunol. (1997) 158:6019–28. 9190957

[50] Weening JJ D'Agati VD Schwartz MM Seshan SV Alpers CE Appel GB. The classification of glomerulonephritis in systemic lupus erythematosus revisited. Kidney Int. (2004) 65:521–30. 10.1111/j.1523-1755.2004.00443.x 14717922

[51] Celhar T Hopkins R Thornhill SI De Magalhaes R Hwang SH Lee HY. RNA sensing by conventional dendritic cells is central to the development of lupus nephritis. Proc Natl Acad Sci USA. (2015) 112:E6195–204. 10.1073/pnas.1507052112 26512111 PMC4653170

[52] Martin RM Brady JL Lew AM. The need for IgG2c specific antiserum when isotyping antibodies from C57BL/6 and NOD mice. J Immunol Methods. (1998) 212:187–92. 10.1016/S0022-1759(98)00015-5 9672206

[53] Fairhurst AM Mathian A Connolly JE Wang A Gray HF George TA. Systemic IFN-alpha drives kidney nephritis in B6.Sle123 mice. Eur J Immunol. (2008) 38:1948–60. 10.1002/eji.200837925 18506882 PMC2699327

[54] Fairhurst AM Wandstrat AE Wakeland EK. Systemic lupus erythematosus: multiple immunological phenotypes in a complex genetic disease. Adv Immunol. (2006) 92:1–69. 10.1016/S0065-2776(06)92001-X 17145301

[55] Lu R Munroe ME Guthridge JM Bean KM Fife DA Chen H. Dysregulation of innate and adaptive serum mediators precedes systemic lupus erythematosus classification and improves prognostic accuracy of autoantibodies. J Autoimmun. (2016) 74:182–93. 10.1016/j.jaut.2016.06.001 27338520 PMC5079766

[56] Zhu J Liu X Xie C Yan M Yu Y Sobel ES. T cell hyperactivity in lupus as a consequence of hyperstimulatory antigen-presenting cells. J Clin Invest. (2005) 115:1869–78. 10.1172/JCI23049 15951839 PMC1143586

[57] Wandstrat AE Nguyen C Limaye N Chan AY Subramanian S Tian XH. Association of extensive polymorphisms in the SLAM/CD2 gene cluster with murine lupus. Immunity. (2004) 21:769–80. 10.1016/j.immuni.2004.10.009 15589166

[58] Wong EB Soni C Chan AY Domeier PP Shwetank Abraham T. B cell-intrinsic CD84 and Ly108 maintain germinal center B cell tolerance. J Immunol. (2015) 194:4130–43. 10.4049/jimmunol.1403023 25801429 PMC4402266

[59] Smith-Bouvier DL Divekar AA Sasidhar M Du S Tiwari-Woodruff SK King JK. A role for sex chromosome complement in the female bias in autoimmune disease. J Exp Med. (2008) 205:1099–108. 10.1084/jem.20070850 18443225 PMC2373842

[60] Souyris M Mejía JE Chaumeil J Guéry JC. Female predisposition to TLR7-driven autoimmunity: gene dosage and the escape from X chromosome inactivation. Semin Immunopathol. (2019) 41:153–64. 10.1007/s00281-018-0712-y 30276444

[61] Jackson SW Scharping NE Kolhatkar NS Khim S Schwartz MA Li QZ. Opposing impact of B cell-intrinsic TLR7 and TLR9 signals on autoantibody repertoire and systemic inflammation. J Immunol. (2014) 192:4525–32. 10.4049/jimmunol.1400098 24711620 PMC4041708

[62] Soni C Wong EB Domeier PP Khan TN Satoh T Akira S. B cell-intrinsic TLR7 signaling is essential for the development of spontaneous germinal centers. J Immunol. (2014) 193:4400–14. 10.4049/jimmunol.1401720 25252960 PMC4201954

[63] Tada Y Kondo S Aoki S Koarada S Inoue H Suematsu R. Interferon regulatory factor 5 is critical for the development of lupus in MRL/lpr mice. Arthritis Rheum. (2011) 63:738–48. 10.1002/art.30183 21305501

[64] De Nardo D Balka KR Cardona Gloria Y Rao VR Latz E Masters SL. Interleukin-1 receptor-associated kinase 4 (IRAK4) plays a dual role in myddosome formation and Toll-like receptor signaling. J Biol Chem. (2018) 293:15195–207. 10.1074/jbc.RA118.003314 30076215 PMC6166714

[65] Marshall JD Heeke DS Gesner ML Livingston B Van Nest G. Negative regulation of TLR9-mediated IFN-alpha induction by a small-molecule, synthetic TLR7 ligand. J Leukoc Biol. (2007) 82:497–508. 10.1189/jlb.0906575 17565046

[66] Marshak-Rothstein A. Toll-like receptors in systemic autoimmune disease. Nat Rev Immunol. (2006) 6:823–35. 10.1038/nri1957 17063184 PMC7097510

[67] Chauhan SK Singh VV Rai R Rai M Rai G. Distinct autoantibody profiles in systemic lupus erythematosus patients are selectively associated with TLR7 and TLR9 upregulation. J Clin Immunol. (2013) 33:954–64. 10.1007/s10875-013-9887-0 23564191

[68] Han S Zhuang H Xu Y Lee P Li Y Wilson JC. Maintenance of autoantibody production in pristane-induced murine lupus. Arthritis Res Ther. (2015) 17:384. 10.1186/s13075-015-0886-9 26717913 PMC4718029

[69] McElroy WT. Interleukin-1 receptor-associated kinase 4 (IRAK4) inhibitors: an updated patent review (2016–2018). Expert Opin Ther Pat. (2019) 29:243–59. 10.1080/13543776.2019.1597850 30916602

[70] Kelly PN Romero DL Yang Y Shaffer AL Chaudhary D Robinson S. Selective interleukin-1 receptor-associated kinase 4 inhibitors for the treatment of autoimmune disorders and lymphoid malignancy. J Exp Med. (2015) 212:2189–201. 10.1084/jem.20151074 26621451 PMC4689168

[71] Hjorton K Hagberg N Israelsson E Jinton L Berggren O Sandling JK. Cytokine production by activated plasmacytoid dendritic cells and natural killer cells is suppressed by an IRAK4 inhibitor. Arthritis Res Ther. (2018) 20:238. 10.1186/s13075-018-1702-0 30355354 PMC6235225

[72] Wang Z Wesche H Stevens T Walker N Yeh WC. IRAK-4 inhibitors for inflammation. Curr Top Med Chem. (2009) 9:724–37. 10.2174/156802609789044407 19689377 PMC3182414

