# Supplementary material for: TLR7 Protein Expression in Mild and Severe Lupus-Prone Models Is Regulated in a Leukocyte, Genetic, and IRAK4 Dependent Manner
Source: Front Immunol. 2019 Jul 10;10:1546. doi: 10.3389/fimmu.2019.01546 (PMC6636428; doi:10.3389/fimmu.2019.01546)
Supplement: Supplementary file 1 [file Data_Sheet_1.pdf]

Supplementary Information for

TLR7 protein expression in mild and severe lupus-prone models is regulated in a leukocyte, genetic and IRAK4 dependent manner.

Teja Celhar, Hao Kim Lu, Lia Benso, Larissa Rakhilina, Hui Yin Lee, Shubhita Tripathi, Olga Zharkova, Wei Yee Ong, Hiroko Yasuga, Bijin Au, Damien Marlier, Lina Hsiu Kim Lim, Thomas Paulraj Thamboo, John S. Mudgett, Matthew F. Mackey, Dennis M. Zaller, John Edward Connolly, and Anna-Marie Fairhurst

Corresponding author: Anna-Marie Fairhurst

Email: annamarie_fairhurst@immunol.a-star.edu.sg

This PDF file includes:

Figs. S1 to S5

Table S1

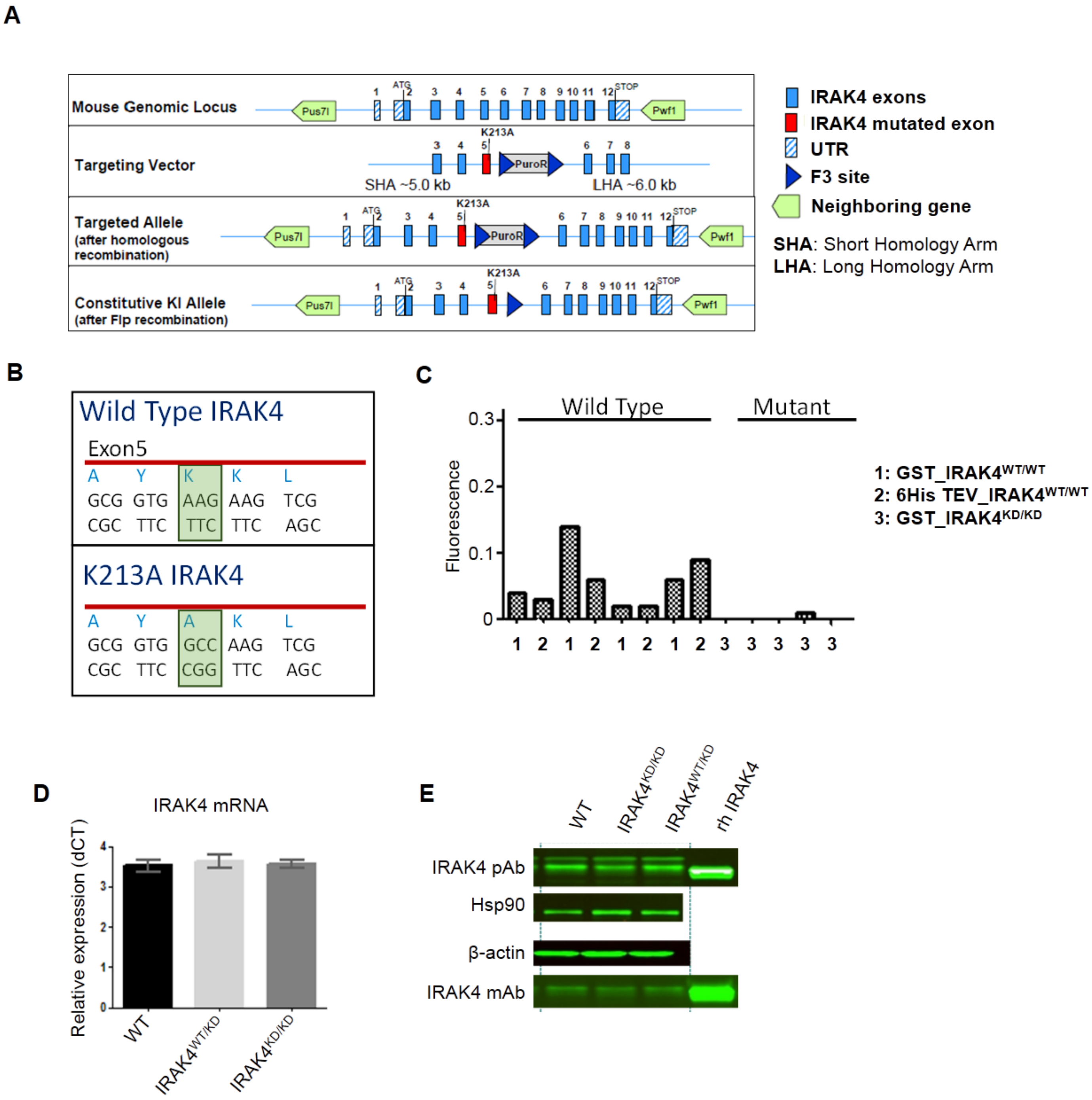

Fig. S1. Generation of IRAK4 K213A mutation. (A) Structure of the mouse IRAK4 gene, targeting vector, targeted allele, and knock in allele. Details are described in the *Materials and Methods*. (B) The resultant sequence substituting alanine for lysine was confirmed by sequencing. (C) GST-IRAK4$^{KD/KD}$ or GST/TEV-IRAK4$^{WT/WT}$ fusion proteins were tested for kinase activity with an ULight assay at 48h. Expression of IRAK4 mRNA (D) and protein (E) in splenocytes of wild type, heterozygous, and homozygous mice. rhIRAK4 was used as a positive control.

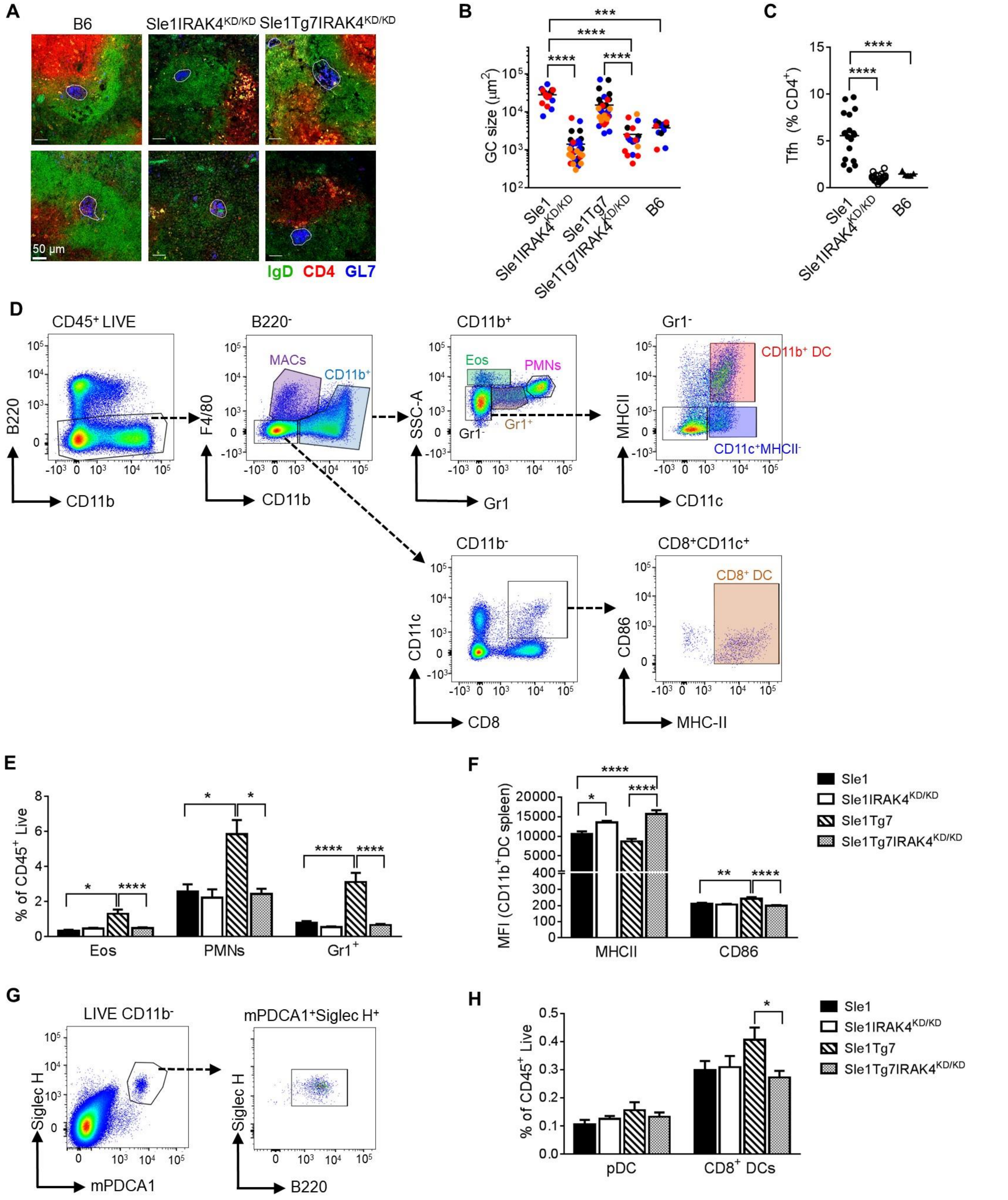

Fig. S2. Effect of IRAK4 kinase dead on disease phenotype in the spleen of Sle1 and Sle1Tg7 mice. Spleens and splenocytes from 6-7 months old *Sle1*, *Sle1*Tg7, *Sle1*IRAK$^{KD/KD}$ and *Sle1*Tg7IRAK$^{KD/KD}$ female mice were analysed by immunofluorescence or flow cytometry, respectively. (A) Representative immunofluorescent staining of germinal centers in splenic sections of from *Sle1*IRAK4$^{KD/KD}$ and

*Sle1*Tg7IRAK4$^{KD/KD}$ mice compared with age-matched B6 mice. Confocal images were taken at 300x manification; anti-GL7 (blue), anti-CD4 (red) and anti-IgD (green). (B) The germinal center area (μm^2) was measured from 12-35 individual GCs from 3-4 mice per group (each mouse is represented by differently coloured dots). (C) The percentages of CD4$^+$ICOS$^+$CXCR5$^+$ T$_{FH}$ from *Sle1* and *Sle1*IRAK4$^{KD/KD}$ mice are compared with aged-matched B6 controls. (D) Representative FACS plots showing the gating strategy for identifying macrophages, eosinophils, PMNs, GR-1$^+$CD11b$^+$ cells, CD11b$^+$ cDC, CD11c$^+$MHC-II$^-$ cells and CD8$^+$ DCs. (E) Cummulative data showing splenic frequencies of eosinophils, PMNs and Gr-1$^+$ CD11b$^+$ cells. (F) Expression of MHCII and CD86 on CD11b$^+$ cDC. (G) Gating strategy to identify splenic pDCs. (H) Frequencies of splenic pDCs and CD8$^+$ DCs. Data shown is from 17-23 mice/group (mean + SEM). Parametric data was assessed by 1-way ANOVA (with Bonferroni's multiple comparisons test) or Student's t test; nonparamentric data by Kruskal-Wallis (with Dunn's multiple comparisons test) or Mann-Whitney test. *$P < 0.05$, **P < 0.01, ***P < 0.001, ****P < 0.0001.

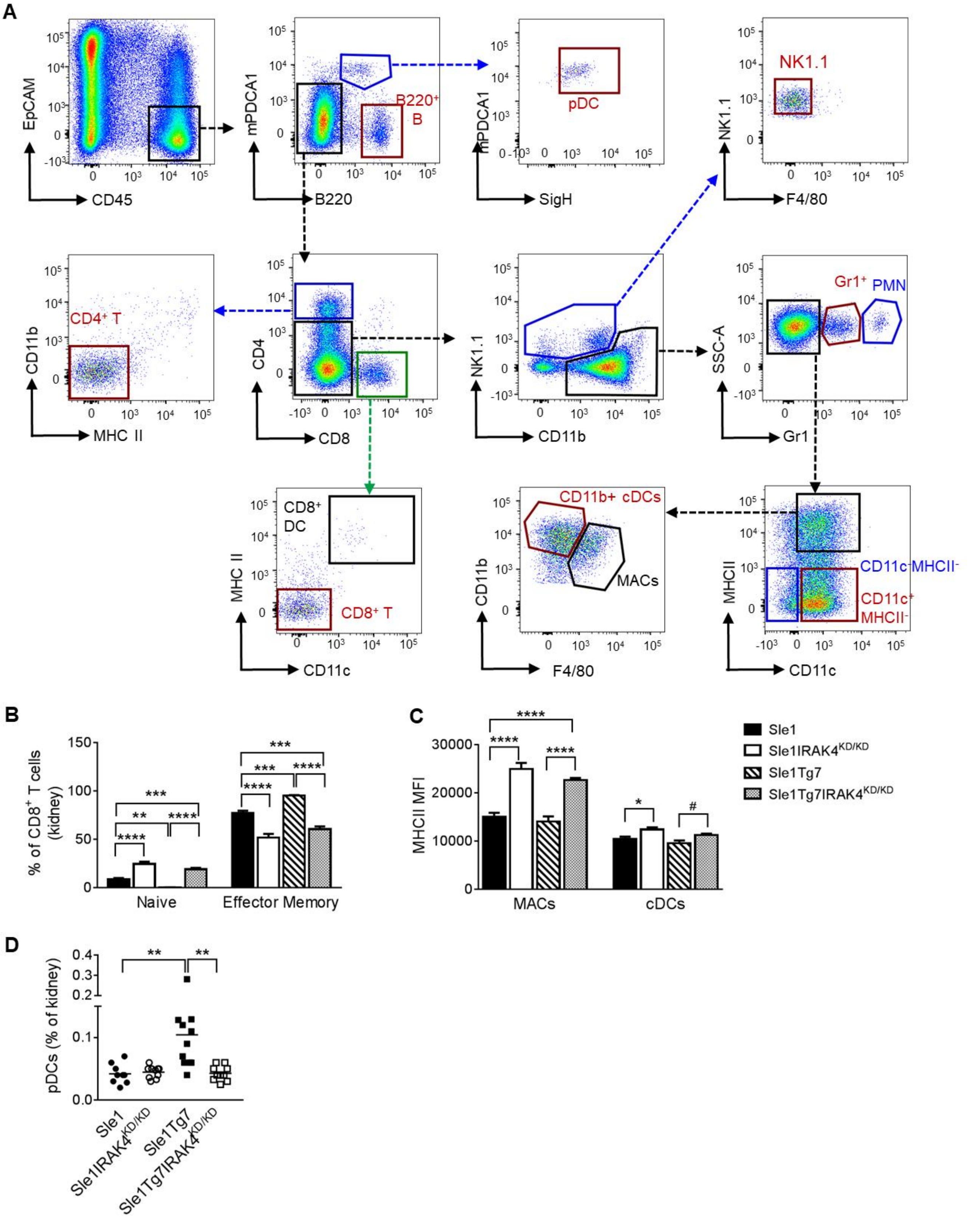

Fig S3. Effect of IRAK4 kinase dead on disease phenotype in the kidneys of Sle1 and Sle1Tg7 mice. Kidneys from 6-7 months old *Sle1*, *Sle1*Tg7, *Sle1*IRAK$^{KD/KD}$ and *Sle1*Tg7IRAK$^{KD/KD}$ female mice were analysed by flow cytometry. (A) Gating strategy used to identify kidney pDCs, B cells, NK1.1^{+} cells, CD4^{+} T cells, CD8^{+} T cells, CD8^{+} DCs, PMNs, Gr1^{+}CD11b^{+} cells, CD11c^{+}MHCII^{-} cells, CD11c^{-}MHCII^{-} cells, macrophages and CD11b^{+} cDCs. (B) Frequencies of naive and memory CD8^{+} T cells in the kidney. (C)

Expression of MHCII on kidney macrophages and CD11b$^+$ cDCs. (D) Frequencies of pDCs in the kidney. Parametric data was assessed by 1-way ANOVA (with Bonferroni's multiple comparisons test) or Student's t test; nonparamentric data by Kruskal-Wallis (with Dunn's multiple comparisons test) or Mann-Whitney test. *$P < 0.05$, **$P < 0.01$, ***$P < 0.001$; ****$P < 0.0001$. # indicates significance between two groups only ($^{\#}P < 0.05$, $^{\#\#\#}P < 0.001$).

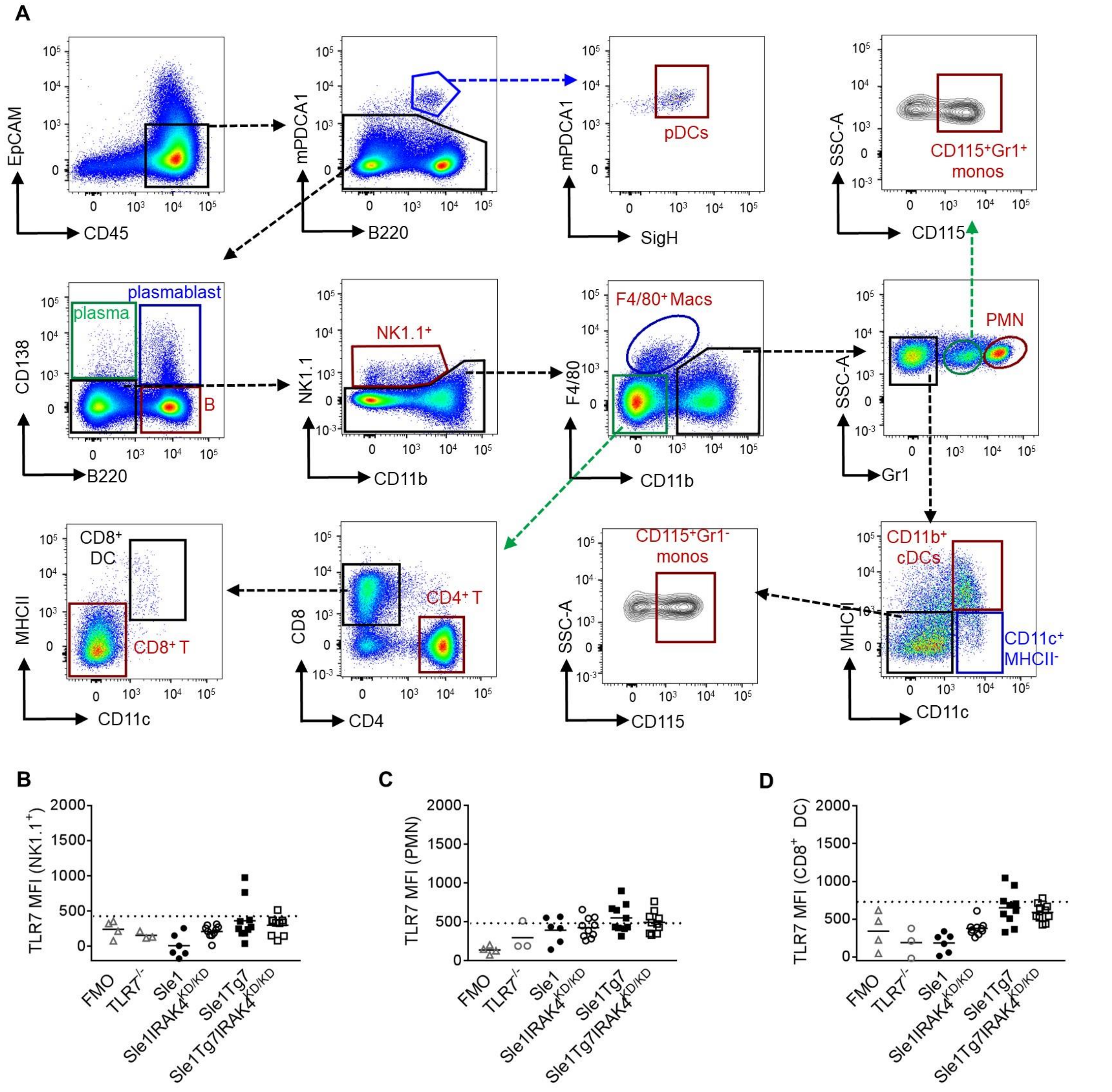

Fig. S4***. Gating strategy and correlation of TLR7 expression in splenocytes.*** (A) Gating strategy for various leukocytes in the spleen. TLR7 expression in splenic (B) NK1.1^{+}, (C) PMNs and (D) CD8^{+} DCs from aged *Sle1*, *Sle1*IRAK4$^{KD/KD}$, *Sle1*Tg7, *Sle1*Tg7IRAK4$^{KD/KD}$ mice. TLR7$^{-/-}$ mice and fluorescent minus one (FMO) controls were used to determine specificity. Dotted line represents mean + 2SD of TLR7$^{-/-}$/FMO values to ascertain positivity. Data is from 6-10 mice per group. Correlations were performed by calculating Pearson's correlation coefficients (r) and their significance assessed by a two-tailed t-test.

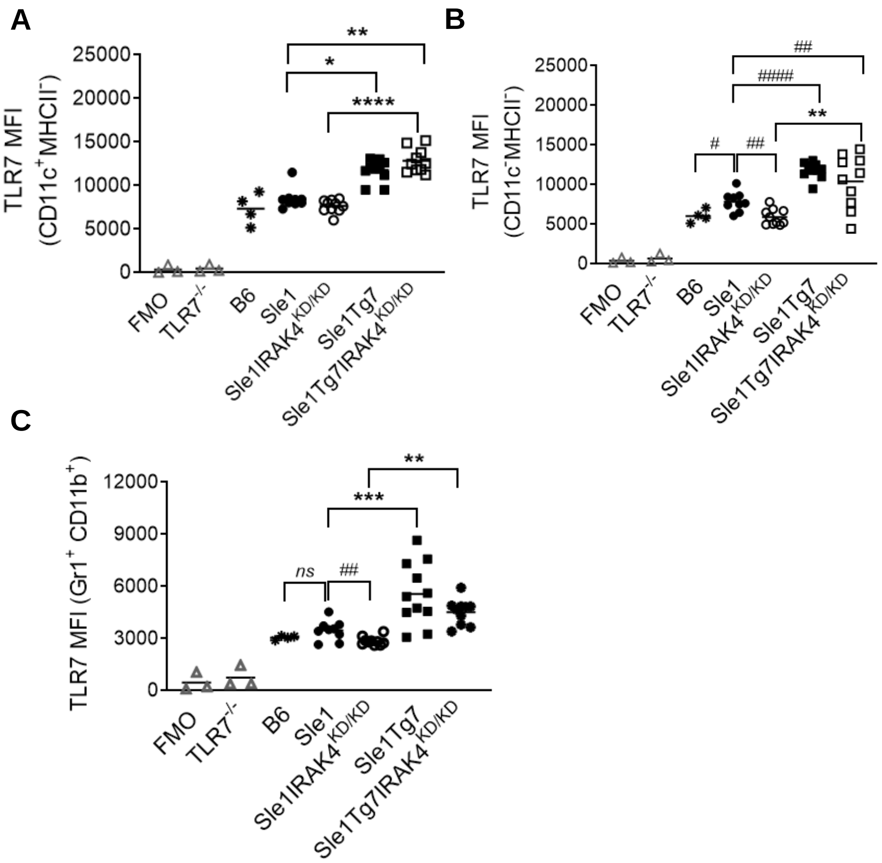

Fig. S5. Expression of TLR7 in kidney leukocytes. TLR7 expression in kidney (A) CD11c^{+}MHCII^{-} (B) CD11c^{-}MHCII^{-} and (C) Gr1^{+}CD11b^{+} cells from the indicated strains. TLR7$^{-/-}$ mice and fluorescent minus one (FMO) controls were used to determine specificity.Data from 9-11 mice per group is shown. Parametric data was assessed by 1-way ANOVA (with Bonferroni's multiple comparisons test) or Student’s t test; nonparamentric data by Kruskal-Wallis (with Dunn's multiple comparisons test) or Mann-Whitney test. $*P < 0.05$, $**P < 0.01$; # indicates significance between two groups only ($^{\#}P < 0.05$, $^{\#\#}P < 0.01$); ns, non-significant.

Table S1. Splenic cell counts

	**Sle1** mean ± STDEV n = 18 – 20	**Sle1IRAK4$^{KD/KD}$** mean ± STDEV n = 20 – 23	**Sle1Tg7** mean ± STDEV n = 13 – 17	**Sle1Tg7IRAK4$^{KD/KD}$** mean ± STDEV n = 19 – 22
**CD19$^+$B220$^+$ B cells (10^6)**	40.26 ± 29.25	33.00 ± 19.08	75.58 ± 60.10	**36.15 ± 15.04$^{\#\#}$**
GC (Fas$^+$GL7$^+$) (10^4)	167.91 ± 141.53	**24.61 ± 21.14*** **	**489.90 ± 495.45$^{\#\#}$**	**23.55 ± 17.95********
MZ (10^4)	729.70 ± 587.24	442.76 ± 208.16	843.21 ± 1218.26	498.44 ± 231.42
Age-related B cells (ABC; CD11b$^+$CD11c$^+$) (10^4)	245.63 ± 136.32	**152.24 ± 80.72$^{\#}$**	**738.82 ± 835.46***	**164.03 ± 106.98********
**Plasma cells (10^4)**	58.11 ± 46.32	23.29 ± 13.92	**224.82 ± 190.15****	**22.95 ± 10.76********
**Plasmablasts (10^4)**	87.48 ± 64.71	65.48 ± 29.60	**203.70 ± 152.78****	**68.82 ± 35.89******
**CD4$^+$ T cells (10^6)**	16.33 ± 8.95	**10.20 ± 4.66$^{\#}$**	**56.85 ± 48.47***	**9.57 ± 2.93********
PD-1 (MFI)	134.02 ± 42.64	**76.38 ± 12.00******	**257.53 ± 97.24$^{\#\#\#\#}$**	**75.66 ± 12.56********
ICOS (MFI)	609.39 ± 204.79	**352.87 ± 112.45****	**1700.94 ± 675.40***	**367.09 ± 127.10********
CD69$^+$ (% CD4$^+$ T)	5.75 ± 2.50	**2.96 ± 1.47****	**12.35 ± 6.27$^{\#\#\#}$**	**2.74 ± 0.93********
Tfh (ICOS$^+$CXCR5$^+$) (10^4)	94.00 ± 63.48	**12.68 ± 9.20********	**448.20 ± 517.77$^{\#\#}$**	**14.14 ± 8.19********
**CD8$^+$ T cells (10^6)**	9.90 ± 5.56	8.70 ± 3.98	14.22 ± 12.00	8.23 ± 2.50
PD-1 (MFI)	89.36 ± 15.21	80.27 ± 11.76	**150.84 ± 47.00****	**79.99 ± 11.63********
ICOS (MFI)	284.11 ± 39.08	280.30 ± 81.90	257.35 ± 86.57	288.91 ± 93.64
**pDC (Siglec H$^+$mPDCA1$^+$) (10^4)**	10.73 ± 13.45	7.80 ± 5.77	**72.96 ± 103.53$^{\#}$**	**8.54 ± 6.85***
CD86 (MFI)	144.21 ± 50.21	148.36 ± 45.13	**92.91 ± 61.33***	**135.83 ± 48.91***
MHCII (MFI)	766.56 ± 384.14	916.08 ± 519.67	1069.75 ± 634.62	1079.34 ± 703.68
CD9$^+$CCR9$^-$ (% pDC)	2.18 ± 1.31	1.33 ± 0.86	**4.89 ± 3.19***	**1.35 ± 1.24********
CD9$^-$CCR9$^+$ (% pDC)	57.68 ± 14.33	59.46 ± 15.25	57.80 ± 11.15	59.08 ± 17.79
**CD8$^+$ DCs (10^4)**	22.38 ± 12.46	**11.32 ± 6.29***	**101.93 ± 108.19****	**14.46 ± 7.31********
**Macrophages (F4/80$^+$CD11b^{low}) (10^4)**	60.43 ± 69.51	35.63 ± 25.63	**410.35 ± 636.46***	**42.42 ± 20.26****
**Myeloid subsets (CD11b$^+$) (10^6)**	6.39 ± 3.56	**2.99 ± 1.83****	**66.02 ± 85.06****	**4.14 ± 1.98********
Eosinophils (10^4)	29.21 ± 31.42	21.15 ±16.05	**403.62 ± 577.33********	**26.21 ± 15.87********
PMNs (10^4)	201.87 ± 175.50	**67.63 ± 54.22***	**1436.75 ± 1672.24****	**132.42 ± 92.87********
CD11b$^+$Gr1$^+$ (10^4)	70.72 ± 52.68	**27.27 ± 21.71***	**1029.4 ± 1413.11******	**40.34 ± 27.47********
CD11b$^+$CD11c$^+$MHCII$^+$ DCs (10^4)	94.89 ± 51.37	**45.37 ± 27.77****	**970.66 ± 1070.17****	**54.02 ± 28.13********
CD11b$^+$CD11c$^+$MHCII$^-$ (10^4)	8.66 ± 6.95	**1.67 ± 1.28****	**729.66 ± 1614.03***	**1.69 ± 1.14********

*$P < 0.05$, **P < 0.01, *** P < 0.001, ****P < 0.0001; assessed among multiple groups by 1-way ANOVA with Bonferroni's multiple comparisons test (parametric data) or by Kruskal-Wallis with Dunn's multiple comparisons test (non-parametric data). P < 0.05, $^{\#\#}P$ < 0.01, $^{\#\#\#}P$ < 0.001, $^{\#\#\#\#}P$ < 0.0001; assessed between two groups with the Student's t test (parametric data) or Mann-Whitney test (nonparametric data). Statistics in the *Sle1*IRAK4$^{KD/KD}$ column are for *Sle1* vs *Sle1*IRAK4$^{KD/KD}$ comparisons, statistics in the *Sle1*Tg7 column are for *Sle1* vs *Sle1*Tg7 comparisons and statistics in the *Sle1*Tg7IRAK4$^{KD/KD}$ column are for *Sle1*Tg7 vs *Sle1*Tg7IRAK4$^{KD/KD}$ comparisons.
